# Inhibition of autophagy in platelets as a therapeutic strategy preventing hypoxia induced thrombosis

**DOI:** 10.1038/s41598-025-91181-y

**Published:** 2025-02-26

**Authors:** Propanna Bandyopadhyay, Yash T. Katakia, Sudeshna Mukherjee, Syamantak Majumder, Shibasish Chowdhury, Rajdeep Chowdhury

**Affiliations:** Department of Biological Sciences, Birla Institute of Technology and Science (BITS Pilani), Pilani Campus, Pilani, Rajasthan 333031 India

**Keywords:** Platelet aggregation, Autophagy, Hypobaric hypoxia, Chloroquine, IVC ligation, Biochemistry, Cell biology, Risk factors

## Abstract

Hypoxia triggers activation of platelets, leading to thrombosis. If not addressed clinically, it can cause severe complications and fatal consequences. The current treatment regime for thrombosis is often palliative and include long-term administration of anticoagulants, causing over-bleeding risk and other secondary effects as well. This demands a molecular understanding of the process and exploration of an alternative therapeutic avenue. Interestingly, recent studies demonstrate that platelets exhibit functional autophagy. This cellular homeostatic process though well-studied in non-platelet cells, is under-explored in platelets. Herein, we report autophagy activation under physiologically relevant hypoxic condition (10% O_2_; associated with high altitude) in *ex-vivo* platelets and in vivo as well. We show that autophagy inhibition using chloroquine (CQ), a repurposed FDA-approved drug, can significantly reduce platelet activation, both in *ex-vivo* and *in-vivo* settings*.* Further, surgical ligation of inferior vena cava (IVC) was performed to induce thrombus formation. Interestingly, CQ pre-treated rats showed reduced clotting ability in surgical animals as well. Importantly, thrombosis inhibitory dose of CQ was considerably lower than the currently used drug-acetazolamide; CQ was also found to be non-toxic to the tissues. Hence, we propose that repurposing of CQ can attenuate hypoxia-induced thrombosis through inhibition of autophagy and can be explored as an effective therapeutic alternative.

## Introduction

Platelets are anucleate cellular fragments that essentially function in maintaining hemostasis and vascular integrity. They are the smallest member among all blood cellular components and actively participate in blood clot formation wherever and whenever necessary^1^. With a very short life span of 7–10 days in humans and 4–5 days in rodents, platelet physiology is quite interesting with limited number of cellular organelles, storage vesicles like granules and lysosomes^2^. Normal platelet functioning involves adhesion, activation, and aggregation of platelets to site of injury. Accordingly, they also possess several proteins on their outer membrane which facilitate their adhesion to either endothelial cells, neighboring platelets or other circulating blood cells upon inflammation or injury^3,4^. These platelet aggregates are bound together by fibrin threads to form outer mesh like structure^4^. Interestingly, just as platelets start adhering, they change their state from ‘inactive’ to ‘active’ simultaneously changing their morphology from discoid to spherical shape, and extending cytoskeletal filaments to have a stronger interaction with the surrounding cells and tissues^5,6^. However, in their inactive state as well platelets have several ongoing cellular processes that help them to maintain homeostasis and keep them upbeat and armed for a necessary transition to the ‘active’ state.

One such homeostatic process of relevance in platelets is autophagy^7^. Importantly, discovery of autophagic machinery is very recent, and subsequent studies further demonstrate its role not only in platelet activation but also in megakaryopoiesis, inflammation and host immune responses^8^. However, given the versatility of platelet functions and already identified multifaceted role of autophagy recognized primarily in nucleated cells, there might be multiple cellular functions regulated by autophagy in the atypical platelet cells. Our understanding toward the same is still at its infancy.

Amongst the diverse cellular functions of autophagy, the most widely accepted is its active involvement in elimination of damaged or unwanted proteins and organelles via engulfment into a vacuolar structure called autophagosome which fuses with lysosomes to degrade the contents. It is induced majorly under stress conditions like nutrient starvation, and chemotherapeutic stress playing a major role in restoring cellular/tissue balance^9,10^. In addition to above, one of the several processes where autophagy and its homeostatic machinery are highly implicated is hypoxia. Autophagy aids in cellular redox and nutrient hemostasis under hypoxic conditions. Herein, a cellular hypoxic state can be defined as disruption in the balance between amount of oxygen required by cells compared to what is acquired. Physiologically this may lead to oxygen tension buildup in arteries due to inability of lungs to properly oxygenate. This state is often associated with various cardiovascular disorders^11^. Deep vein thrombosis (DVT) is one such condition that occurs as a lowlander travels to a higher altitude and encounters a hypoxic state^12^. This results in unnecessary clot formation called thrombus, which may initially occur in the calf muscles, can subsequently travel throughout the body via blood vessels resulting in a state known as venous thromboembolism (VTE). Occasionally, such clots can reach vital organs like lungs and heart blocking the movement of blood (pulmonary thromboembolism) causing serious conditions like myocardial infarction and stroke^13,14^.

Importantly, since hypoxia can activate platelets and autophagy as well, in this study we planned to dissect the link between them. Though there are a few studies demonstrating autophagic status in platelets, yet the role of autophagy inhibition on platelet functionality under hypoxic condition has not yet been explored. Our study not only provides insights into the role of autophagy in platelet activation and thrombosis under low oxygen conditions *ex-vivo* and *in-vivo*, but also offers a novel therapeutic alternative for prevention of hypoxia-induced thrombotic clots.

## Results

### Hypoxia (10%) induces pro-thrombotic features in platelets

**Fig. 1:**
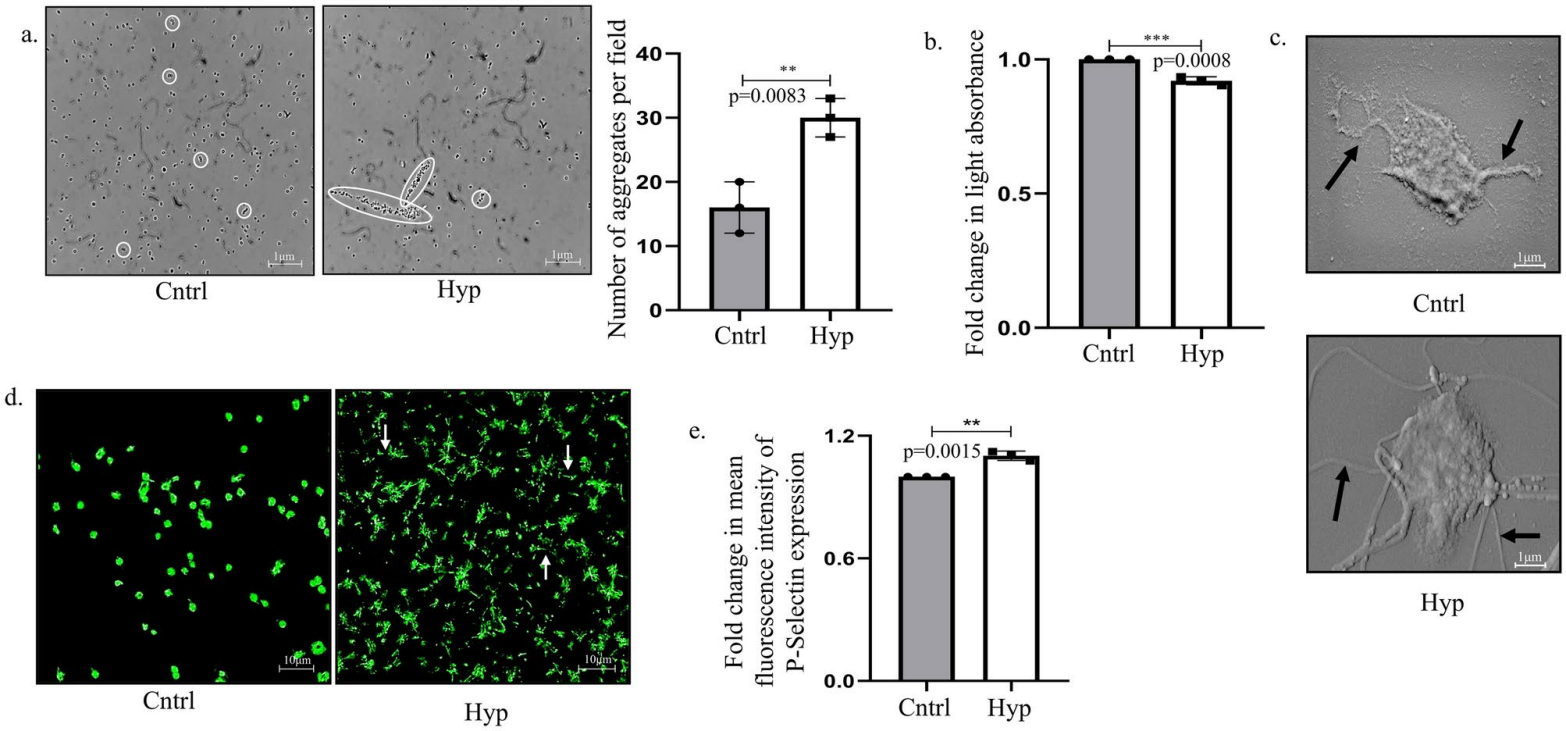
Hypoxia (10%) induces pro-thrombotic features in platelets. (**a**) Phase-contrast microscopic images and graphical representation of platelet aggregates under normoxic (Cntrl; 21% oxygen) or hypoxic condition (Hyp; 10% oxygen; 30 min exposure) (Scale bar: 1 μm). (**b**) Graphical representation of light transmission aggregometry showing fold change in light absorbance owing to exposure to normoxia or hypoxia. (**c**) Scanning electron microscopy (SEM) images of platelets showing cytoskeletal extensions from platelet surface (denoted by black arrows) under hypoxia compared to normoxia (50,000 × magnification). Additional acquisition details of SEM images are provided in the ‘equipment and settings’ Section. (**d**) Phalloidin staining of cells depicting filamentous bridges (denoted by white arrows) formed between platelets under low oxygen conditions (10% oxygen for 30 min) (Scale bar: 10 μm). Details of image acquisition settings are mentioned in the ‘equipment and settings’ section. (**e**) Mean fluorescence intensity of P-Selectin as analyzed by flow cytometry 30 min post-exposure to 21% oxygen (Cntrl) or 10% oxygen (Hyp). **p < 0.01, ***p < 0.001.

Acute hypoxic conditions are reported to induce a pro-thrombotic state in platelets; however, effect of a physiologically relevant hypoxic state associated with high altitude is poorly understood. To evaluate the same, platelets were isolated by retro-orbital bleeding from female Wistar rats and were cultured *ex-vivo* under normoxic (21%O_2_) or hypoxic conditions (10%O_2_). Unlike majority of the existing studies, herein a physiologically relevant oxygen percentage (10%O_2_) that is often experienced at high altitudes in recreational climbers or soldiers was considered over an acute hypoxia^15,16^. As evident from phase contrast images and correlated quantitative bar diagram, an increased aggregation of platelets was observed under hypoxia (Fig. 1a). In addition, we performed a light transmission aggregometry experiment, as described by Chan et al., which states that light absorbed by platelets is inversely related to formation of aggregates^17^. Interestingly, a significantly (*p valu*e 0.0008) reduced light absorbance, putatively attributed to aggregation of platelets under hypoxic condition was observed (Fig. 1b). Furthermore, abundant cytoskeletal extensions, indicative of activated platelets could be clearly visualized in scanning electron microscopic (SEM) images (Fig. 1c)^6,18^. Formation of cytoskeletal protrusions after phalloidin staining of platelet actin filaments as evident from extensive green projections under hypoxia also confirmed altered platelet homeostasis (Fig. 1d). The above results are indicative of the fact that a non-acute-hypoxic condition, like 10%O_2_ can also stimulate a state reflective of activated platelets *ex-vivo*. Herein, existing literature suggests that platelets demonstrate an increased adhesive property upon appropriate stimuli to facilitate their clot formation^18^. Interestingly, a 10%O_2_ exposure also stimulated an increased adhesion of cultured platelets onto collagen-coated surfaces (Supplementary Fig. 1a). Clot retraction assay is often used as a reproducible, alternate approach to effectively assess platelet activation^19,20^. Qualitative estimation of formed clot under hypoxia showed that the mesh gets substantially retracted compared to normoxia, indicative of clotting (Supplementary Fig. 1b). Platelet activation is conventionally monitored at the molecular level through analysis of P-Selectin which is generally stored in alpha granules of platelets and expressed on its membrane surface upon activation. Importantly, P-Selectin protein levels were found to be increased under hypoxic condition, when compared to normoxia, as analyzed through flow cytometry (Fig. 1e). The above results demonstrate the platelet adhesion and activation potential of 10%O_2_ when cultured *ex-vivo*; however, the key molecular signals driving it remains to be further elucidated.

### Exposure of platelets to 10% hypoxia triggers autophagy

**Fig. 2:**
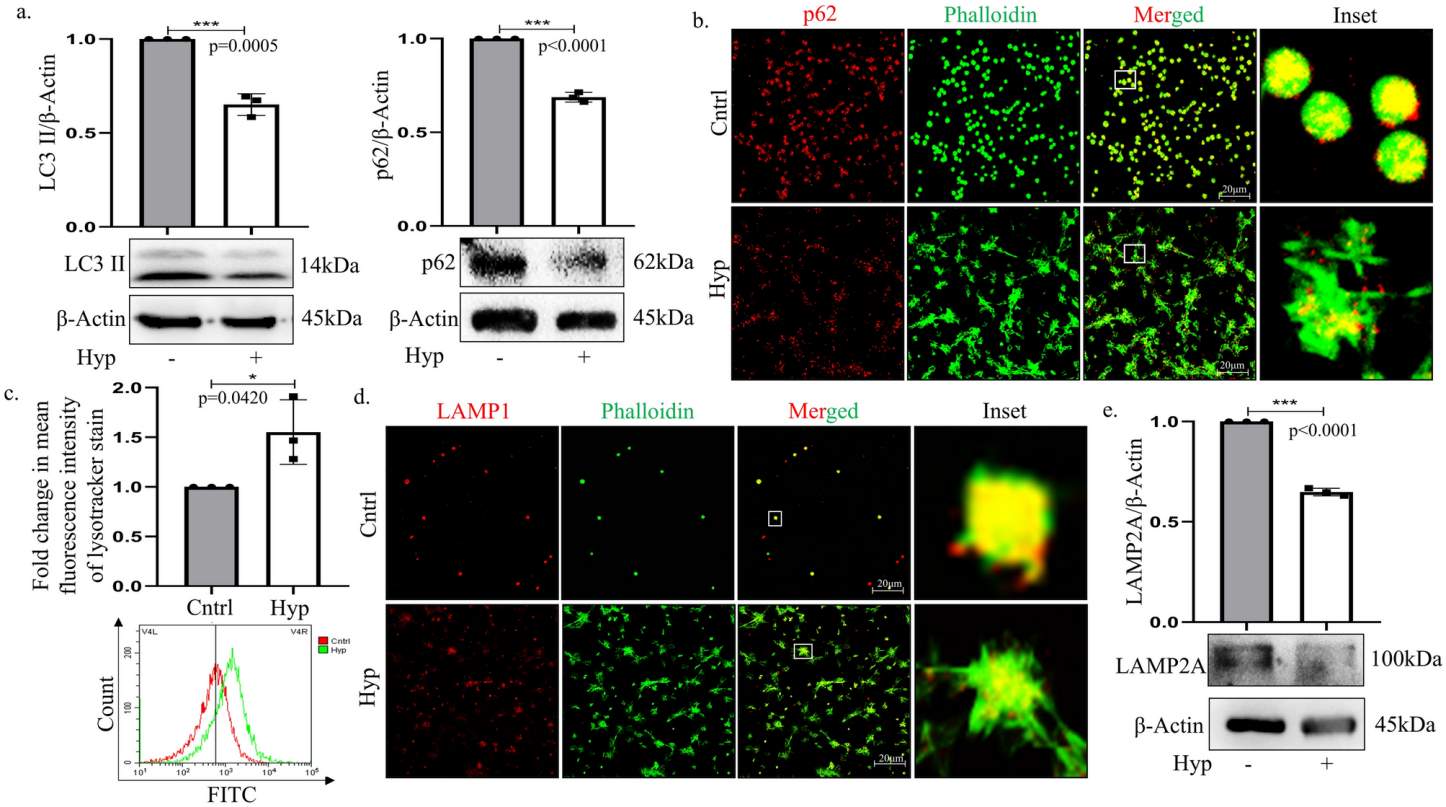
Hypoxia induces autophagy in platelets. (**a**) Immunoblot of autophagy-associated proteins LC3II and p62 in platelets exposed to normoxia or hypoxia for 30 min. (**b**) Immunofluorescence of p62 protein (Red) in platelets upon subjecting them to hypoxia (30 min) compared to normoxia. Cells were co-stained with phalloidin (Green) (Scale bar: 20 μm). (**c**) Graphical representation of lysosomes in platelets under hypoxia measured with lysotracker by flow cytometry. (**d**) Immunofluorescence of LAMP1 (Red) in platelets exposed to varying oxygen conditions- Normoxia (Cntrl; 21%) or Hypoxia (Hyp; 10%) for 30 min. Cells were co-stained with phalloidin (Green) (Scale bar: 20 μm). Inset depicts zoomed image. Details of image acquisition settings are mentioned in equipment and settings Section. (**e**) Immunoblot of LAMP2A expressed by platelets when exposed to hypoxia for 30 min. All the original uncropped blots are provided in the supplementary Fig. 5. *p < 0.05, ***p < 0.001.

Recent literature indicates that cellular structures associated with autophagy and its degradative function are present in platelets and may play a key role in its associated functions like clotting, inflammation or immunity. Importantly, though autophagy is well-studied in nucleated cells, its function in anucleate platelet cells is way less recognized. Moreover, since platelets are cells without nuclear machinery to continuously synthesize new mRNAs, tracking autophagy in these atypical cells is tricky. We hence followed methods from Madhu M Ouseph et.al. 2015 who proved that a reduction in LC3-II can serve as a clear indicator of autophagy in both activated (with thrombin) murine and human platelets^8^. On a similar note, in a separate study Lee et al. reported that the adapter protein SQSTM1/p62 undergoes degradation in activated human platelets and reduced p62 protein conventionally serves as autophagy marker^21^. In corroboration to above, we also observed a decline in both LC3-II and p62 under 10%O_2_ confirming active autophagy (Fig. 2a). There was a simultaneous decrease in fluorescence intensity of p62 protein in platelets exposed to hypoxia as compared to normoxia (Fig. 2b). Interestingly, our flow cytometric analysis revealed significant increase in lysotracker fluorescence post hypoxia exposure further validating a putatively active autophagic flux in the platelets (Fig. 2c). Herein, lysosomes serve as degradation hub for autophagosomal or endocytic vesicles and the lysosomal associated membrane protein 1/2 (LAMP1/2) is known to be distributed amongst these heterogenous endocytic, autophagic and endolysosomal structures^22^; thus, reduction in protein level of LAMP1/2 can often serve as an indicator of active autophagic flux^23^. As published by Monaci et. al. in 2022, hypoxia increases acidity in dendritic cells causing increase in lysotracker fluorescence along with reduced LAMP1 intensity^24^. Importantly, we also observed a reduced fluorescence intensity of LAMP1 in platelets exposed to 10%O_2_ along with decrease in LAMP2A protein expression (Fig. 2d,e)^25^.

### Chloroquine inhibits hypoxia induced autophagy in platelets ex-vivo

**Fig. 3:**
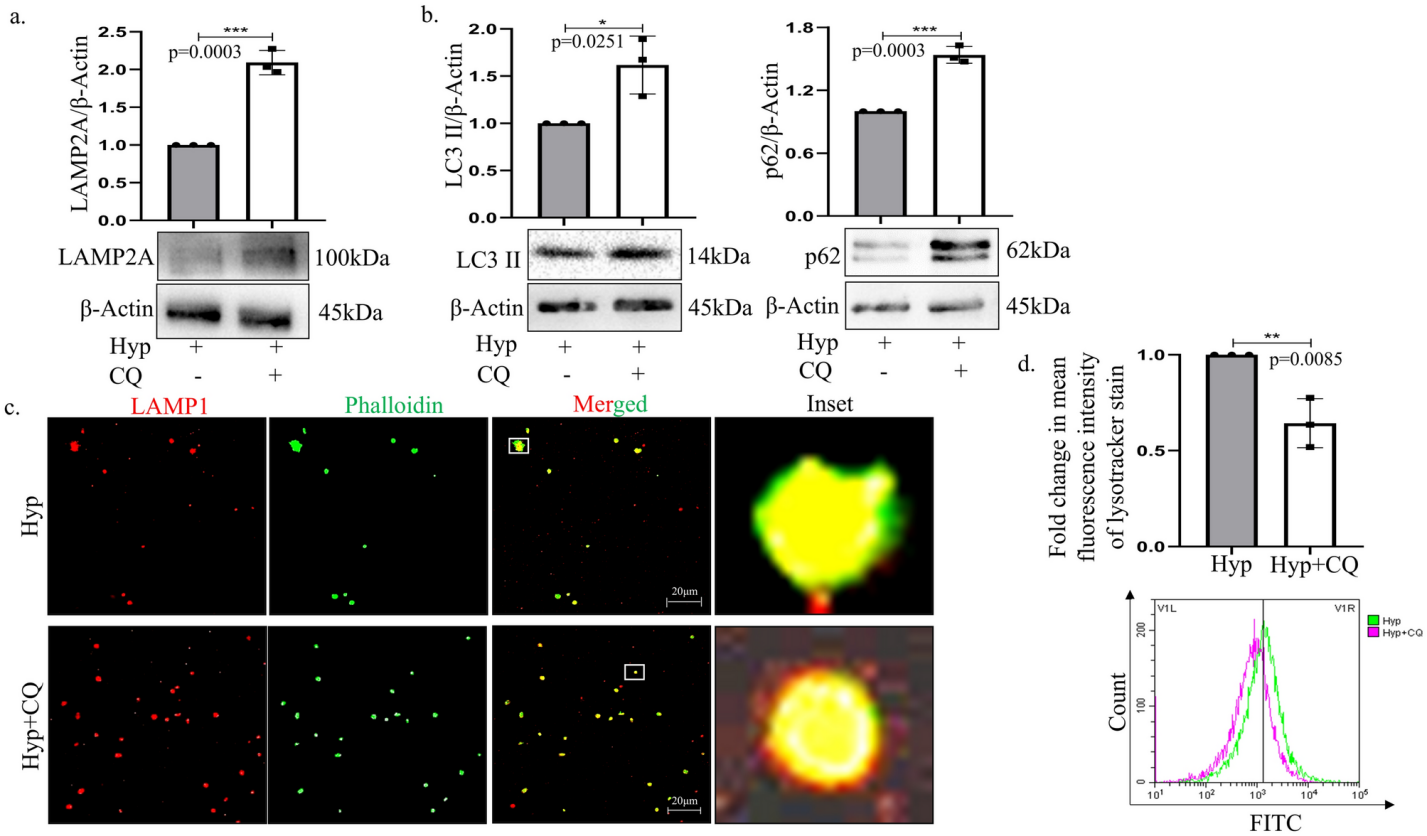
Chloroquine inhibits platelet activation. (**a**) Immunoblot of LAMP2A in platelets exposed to either hypoxia (10% oxygen; Hyp) or hypoxia plus chloroquine (CQ; 2 μM) for 30 min. (**b**) Immunoblot of autophagy-associated markers-LC3II and p62 in platelets treated with or without CQ under hypoxia for 30 min. All the original uncropped blots are provided in the Supplementary Fig. 7. (**c**) Immunofluorescence of LAMP1 (Red) in platelets subjected to CQ and hypoxia post 30 min exposure. Cells were co-stained with phalloidin (Green) (Scale bar: 20 μm). Details of image acquisition settings are mentioned in the ‘equipment and settings’ Section. (**d**) Fold change in lysotracker staining in platelets when exposed to hypoxia in presence or absence of CQ. *p < 0.05, **p < 0.01, ***p < 0.001.

Given that autophagy has earlier been described for its role in platelet activation and our current findings also indicate that hypoxia stimulates autophagy in platelets, we therefore decided to inhibit the homeostatic process with existing drugs. In this regard, the current practice is to impair autophagy by blocking the lysosomal degradation process with compounds like, bafilomycin (Baf) or chloroquine (CQ) or protease inhibitors^26^. Amongst the above, CQ and HCQ (hydroxychloroquine) are FDA-approved, have been widely repurposed, and are hence the primary choice for use in clinics against various diseases. CQ disrupts the binding of autophagosomes with lysosomes and can have non-autophagic effects as well like- disorganization of the cytoskeleton or Golgi/endo-lysosomal organization^26^. Therefore, we exposed platelets to CQ under both normoxic and hypoxic conditions and analyzed its cytotoxic and autophagy inhibitory potential on platelets. As evident from the Supplementary Fig. 1c the specific dose of CQ (2 μM) used for subsequent study had a minimal cytotoxic effect on the platelets. Importantly, CQ treatment resulted in increased accumulation of proteins like LAMP2A, LC3II and p62 in platelets as compared to only hypoxic condition, analyzed by immunoblot (Fig. 3a,b). Furthermore, immunofluorescence analysis showed accumulation of red fluorescence representative of LAMP1 and p62 elevation in platelets exposed to CQ implicating buildup of vesicles positive for these markers due to disrupted cytoskeletal trafficking (Fig. 3c and supplementary Fig. 1d). In addition, exposure of platelets to lysotracker that is known to stain acidic lysosomal compartments also depicted reduced fluorescence. CQ has been earlier reported to alter the lysosomal pH thus interfering with autophagosome-lysosome fusion^27^; the same was found to be true for atypical cells like platelets as well (Fig. 3d). Therefore, we hypothesized that FDA-approved drug CQ can be an excellent choice to inhibit autophagy in platelets to modify their function under hypoxic condition, which was further explored in this study.

### Chloroquine attenuates hypoxia-induced platelet activation ex-vivo

**Fig. 4:**
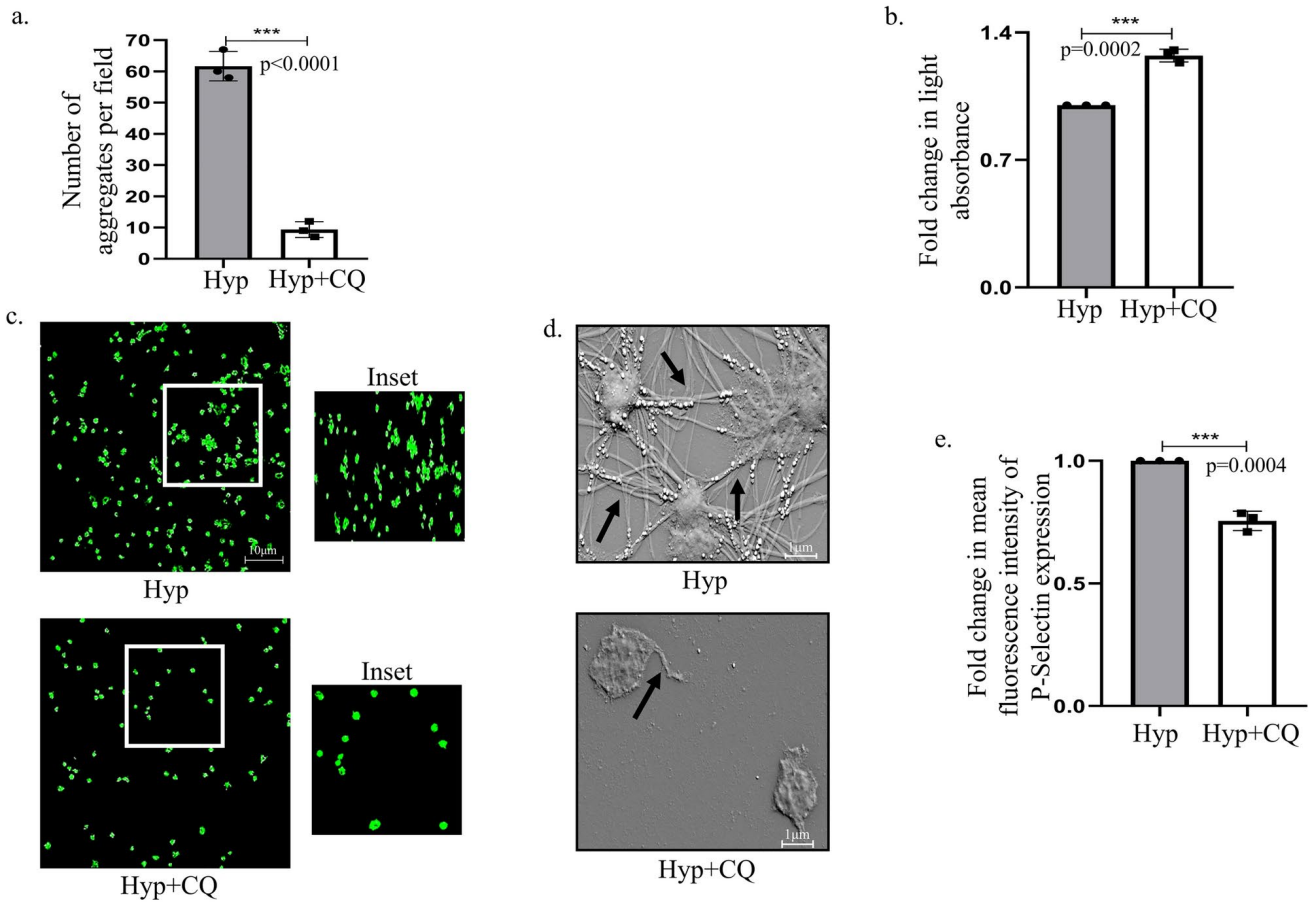
Chloroquine attenuates hypoxia-induced platelet activation *ex-vivo*. (**a**) Images and bar graph showing platelet aggregates under hypoxia (10% oxygen; 30 min) in presence or absence of CQ (2 μM) (Scale bar: 1 μm). (**b**) Graphical representation of light absorbed by platelets under hypoxia (Hyp) in presence or absence of CQ (Hyp + CQ). (**c**) Phalloidin staining of cytoskeletal filaments in platelets exposed to CQ under hypoxia (Scale bar: 10 μm). Details of image acquisition settings are mentioned in the ‘Equipment and settings’ section. (**d**) Scanning electron microscopy of platelets after CQ treatment under hypoxia (30,000 × magnification). Black arrows represent observed filamentous protrusions. Additional acquisition details are provided in the ‘Equipment and settings’ Section. (**e**) Graphical representation of fluorescence intensity of platelet activation mark-P-Selectin under hypoxia in presence or absence of CQ, analyzed through flow cytometry. ***p < 0.001.

To understand the effect of CQ on platelet function, we pre-treated the platelets before exposure to hypoxia. Interestingly, a significant decrease of platelet aggregates was observed under phase-contrast microscope in presence of CQ (Fig. 4a). On a similar note, light transmission aggregometry also demonstrated an increase in light absorbance by platelets with CQ reflective of a decreased platelet aggregation (Fig. 4b). In addition, phalloidin staining showed a decreased cytoskeleton-mediated platelet-platelet interaction suggesting that autophagy inhibition can have a negative impact on platelet aggregative properties induced by hypoxia (Fig. 4c). Platelets upon activation reorganizes its actin cytoskeleton resulting in formation of spiky filamentous structures^28^. Interestingly, scanning electron microscopy clearly showed a drastic reduction of hypoxia-induced cytoskeletal extensions upon autophagy inhibition (Fig. 4d). Further, the clot retraction assay post CQ treatment depicted expansion of mesh, supporting the potential of CQ in reducing clot formation (Supplementary Fig. 1e). In parallel, isolated platelets were treated with acetazolamide (100 μM), a commonly used anti-thrombotic agent, and its effect was compared with CQ in attenuating the pro-coagulant effect of hypoxia^29^. Herein, it was observed that acetazolamide (100 μM) inhibited platelet aggregation at a comparatively higher dose compared to CQ (2 μM), inferring that the latter is putatively more efficacious (Supplementary Fig. 2a). Proteins stored in platelet alpha-granules play a significant role in the identification of platelet activation status. As expected, there was an attenuation of P-Selectin levels upon autophagy inhibition (Fig. 4e). Thus, from the above experiments, it can be inferred that autophagy plays a positive role in platelet activation under hypoxic condition, and its inhibition can potentially lower clot formation.

### Chloroquine inhibits hypoxia-induced platelet activation in-vivo

**Fig. 5:**
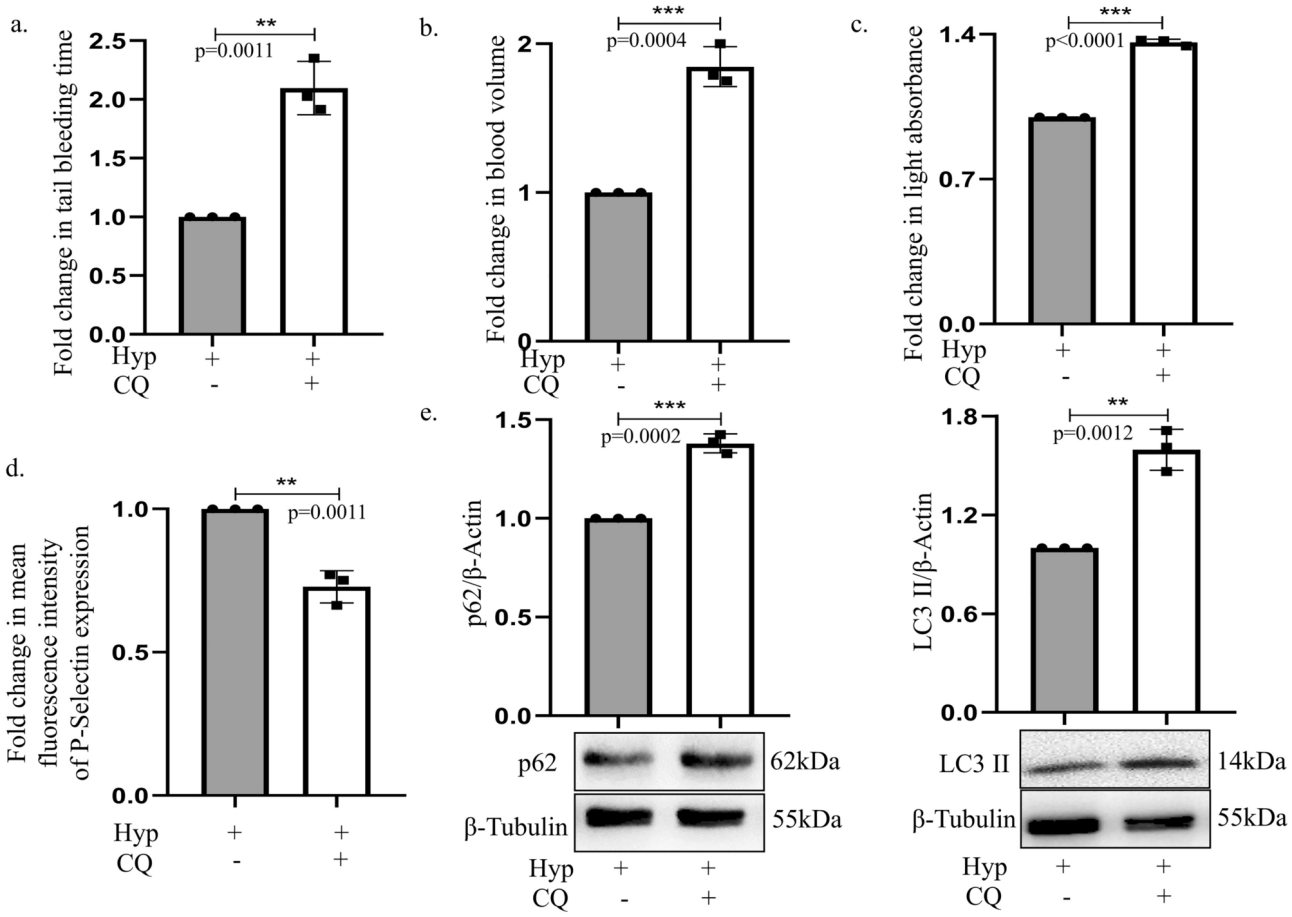
Chloroquine inhibits platelet activation *in-vivo*. (**a**) Graphical representation of fold change in bleeding time in CQ pre-treated (5 mg/kg body weight; intraperitoneal; 30 min) hypoxia-exposed animals (10% oxygen; 24 h). (**b**) Graphical representation showing fold change in blood volume collected from CQ-treated animals under hypoxic condition. (**c**) Graphical representation of light transmission aggregometry based fold change in light absorbance in platelets obtained from *in-vivo* conditions. (**d**) Graph representing fold change in fluorescence intensity of P-Selectin in rats exposed to hypoxia (24 h) pre-injected with intra-peritoneal CQ. (**e**) Immunoblot of p62 and LC3II expressed by *in-vivo* derived platelets when rats were subjected to hypoxia for 24 h post intra-peritoneal CQ injection. All the original uncropped blots are provided in the Supplementary Fig. 10. **p < 0.01, ***p < 0.001.

To simulate a closer life-mimicking scenario we inhibited autophagy *in-vivo*. Herein, hypoxia is reported to act as an inducer increasing platelet count and activation under *in-vivo* conditions^27^. Thus, to explore the potential of CQ under physiologically relevant hypoxic condition, animals were pre-treated with CQ and subjected to 10%O_2_ for 24 h in a hypoxia chamber, constructed in-house for hypoxia treatment^30,31^. Interestingly, an increased bleeding time (tail bleeding experiment) and enhanced blood volume was observed with CQ treatment as compared to hypoxic control animals (Fig. 5a,b) conclusive of the fact that time required for clotting increases with CQ treatment under 10%O_2_. Further, blood samples were collected from these animals to explore platelet activation. To achieve this, light transmission aggregometry was carried out and results showed increase in light absorbance indicative of decreased platelet aggregates (Fig. 5c). Furthermore, reduced P-Selectin expression with CQ confirmed decrease in platelet activation *in-vivo* (Fig. 5d). In accordance to the above results, an increase in p62 and LC3 expression was found in platelet samples isolated post hypoxia and CQ treatment confirming an autophagy inhibition (Fig. 5e). In addition, whole cell proteomic analysis (of platelets isolated from rats) was performed to have a profile of proteins de-regulated under only hypoxia, or CQ with hypoxia exposure. Importantly, in corroboration to our earlier findings, a de-regulation in expression pattern of proteins associated with platelet adhesion, activation, aggregation, and also autophagy was observed under hypoxia; a list of some of the proteins de-regulated and involved in the above processes is presented in the Supplementary Fig. 2b. Interestingly, for example, integrin-linked protein kinase (ILK) which is known to play a major role in initiation of thrombus formation, and is also positively implicated in autophagy, was found to be expressed in hypoxia but was absent in CQ-treated group^32,33^. Further research is required in this direction to dissect the role of such individual proteins in the above context. Finally, to ascertain that CQ dose administered has minimal cytotoxic effect on cellular tissues, histological sectioning of liver and kidney was performed after CQ treatment. Importantly, it did not show any significant aberrations or cytotoxic effect suggesting that CQ, at the stipulated dose was well tolerated and had minimal effect on the tissue integrity (Supplementary Fig. 2c,d). All the above observations from both ex*-vivo* and *in-vivo* systems suggest that CQ can be used as a putative alternative to compromise platelets’ activity and thus thrombotic clot formation through inhibition of autophagy under low-oxygen conditions.

### Chloroquine causes decrease in thrombus formation in flow restriction model

**Fig. 6:**
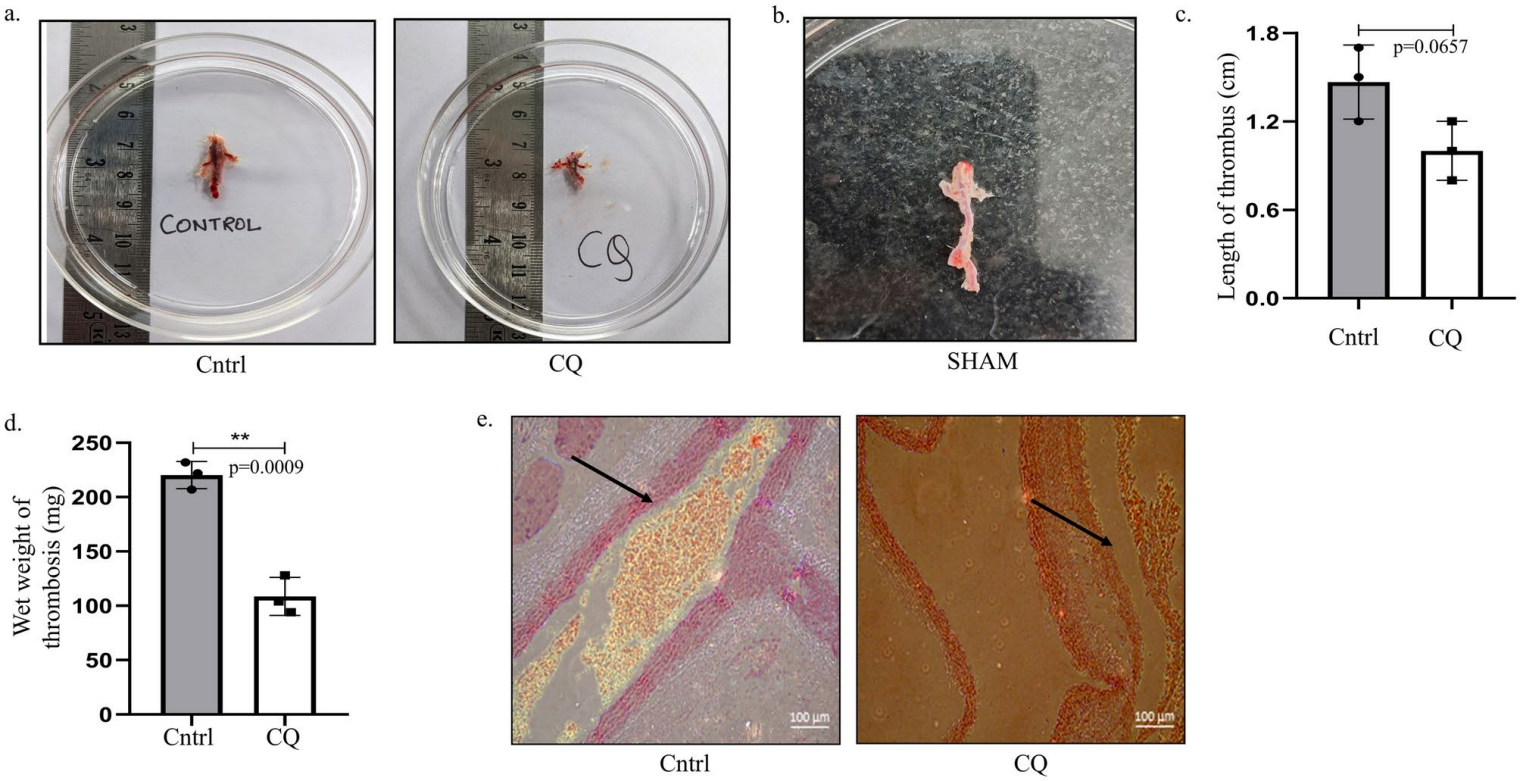
Autophagy inhibition causes decrease in thrombus formation in flow restriction model. (**a**) Thrombus images collected 24-h post ligation of inferior vena cava (IVC) with or without CQ pre-treatment (5 mg/kg body weight; intraperitoneal; 30 min). (**b**) IVC region collected from SHAM animal (non-ligated) model post 24-h of surgery showing absence of thrombus in it. (**c**) Graphical representation of thrombus length (cm) obtained post-surgery from CQ-treated or untreated animals. (**d**) Graphical representation of wet weight of isolated thrombus from CQ-treated or untreated animals. (**e**) Histology of IVC showing blood vessel blockage in CQ untreated ligated animals, compared to a clean vessel obtained post-24-h surgery with CQ treatment (Scale: 100 μm). **p < 0.01.

To finally confirm our results obtained above, a flow restriction model was developed by ligating the inferior vena cava (IVC) and its lateral tributaries to initiate thrombus formation in rats. This ligation has already been proven in earlier studies as a source of rapid venous thrombi generator which can induce thrombus formation as early as 15 min post-surgery^34^. Herein, the ligation of blood vessels leads to restricted blood flow causing platelets to get activated and form thrombus. This surgery was performed to simulate a thrombosis that might occur *in-vivo* at high altitude^30^. Interestingly, if CQ was administered 30 min before surgery, a reduced thrombus size was observed in surgical animals, compared to non-treated animals, as evident from thrombus image (Fig. 6a). A clean IVC observed in SHAM control animals confirmed that thrombus formation is initiated by surgical restriction of blood flow and not by abdominal incision (Fig. 6b). Upon measurement of thrombus size and wet weight, a reduction in both the parameters was evident upon surgical restriction coupled to CQ-exposure compared to control or non-inhibition (Fig. 6c,d). Finally, histology of isolated thrombus confirmed a blockage in blood vessels, as indicated by black arrow in untreated animals, whereas a clearer blood vessel was observed in CQ-treated animals (Fig. 6e). Thus, these results affirm that CQ effectively inhibits autophagy in flow-restricted animal models as well, causing decrease in thrombus size and hence can be a potential arsenal attenuating thrombosis.

## Discussion

Autophagy is a cellular homeostatic mechanism that regulates energy distribution in our body by engulfing and eliminating damaged and unwanted organelles and/or proteins and lipids of cells. Its importance during starvation, disease progression, and other stress responses is quite well known. Interestingly, a recent article by Feng et.al. reported the presence of autophagy machinery and its key role in platelet function^35^. Therefore, platelets-despite being cellular fragments shredded during megakaryocyte maturation, possessing limited life span, restricted cellular machinery to sustain, have mechanisms like autophagy that can regulate homeostasis. In accordance to above, in our study, we observed that autophagy is up-regulated and plays a significant role in governing platelet function upon exposure to physiologically relevant hypoxic condition. A low oxygen condition triggered translation of autophagic mRNAs presumably pre-present in resting platelets, leading to platelet activation and turning on their aggregation- resulting in a thrombotic condition. We evaluated the involvement of autophagy in medicating this process and validated it in both *ex-vivo* and *in-vivo* conditions under low oxygen conditions. Earlier, Lee et.al. reported link between the two processes, however, our group is arguably the first to show an association between the cellular homeostatic process autophagy and the hemostatic process under 10%-oxygen levels^21^. These results are of immense relevance as various physical activities like recreational climbing, training, pregnancy and sudden movement of low-landers to high altitude are reported to induce such physiological low oxygen conditions predisposing a healthy individual to an abrupt state of medical attention. While hypoxia-regulating proteins have been explored as therapeutic targets against such thrombotic tendencies, we for the first time propose the use of chloroquine (CQ) as a therapeutic alternative^12^. CQ is an FDA-approved drug currently marketed as an anti-malarial agent and is presently undergoing clinical trial along with hydroxychloroquine as an autophagy inhibitor for tumor treatment^26^. Its role as autophagy inhibitor in platelets and subsequently in modifying thrombosis under physiologically relevant hypoxic condition has not been evaluated earlier. Herein, we show through *ex-vivo*, *in-vivo* and ligation models that CQ can effectively inhibit autophagy and platelet function as illustrated in graphical abstract. In addition, total proteomic analysis from CQ-exposed rats also confirmed platelet inhibitory activity of CQ. Importantly, the conventional therapeutic strategy for thrombosis includes the use of marketed formulations such as anti-thrombotic agents- acetazolamide, warfarin, dabigatran, edoxaban, aspirin, etc. which often requires either a very high dose or a long-term administration and also might carry the disadvantage of blood thinning causing elevated blood loss post-injury. Therefore, our study is a therapeutic breakthrough depicting not only the role of autophagy in governing platelet functionality under clinically relevant hypoxic conditions but also investigates the therapeutic effects of CQ-an already approved drug that can therefore be re-purposed saving time, money, and efforts required towards introduction of a novel therapy. In addition, autophagy being an integral part of platelet life span starting from its differentiation from megakaryocytes to its activation^36–39^, the fundamentals of how specific autophagic proteins regulate platelet activation shall be very interesting to dissect in the future years to come.

## Materials and methods

### Chemicals and reagents

Trisodium citrate (Na_3_C_6_H_5_O_7_ #20242) and Potassium chloride (KCl, #39594) were acquired from s d fine-chem limited. Citric acid (C₆H₈O₇ #22585) was obtained from Fisher Scientific. Sodium chloride (NaCl, #1.93606.5021), Sodium dihydrogen phosphate (NaH_2_PO_4_ #61784505001730) and Diethyl Ether (#1.00923.2521) was procured from Merck. Magnesium chloride (MgCl_2_, #1349130) and thiazolyl blue tetrazolium bromide (MTT, #33611) were purchased from SRL. HEPES sodium salt (#TC066-25G) and Gelatin (#TC041-500G) were purchased from Himedia. Chloroquine (CQ, #C6528), Acetazolamide (#A6011), Phenylmethylsulfonyl fluoride (PMSF, #P7626), Thrombin (#T4393-100UN) and Dextrose (C₆H₁₂O₆ #G7021) were obtained from Sigma. Thermo Fisher Scientific was approached for obtaining lysotracker green DND-26 (LTG, #L7526), and Collagen (#A1048301). Phalloidin-iFluor 488 reagent (#Ab176753) was purchased from Abcam. Enhanced Chemiluminescence (ECL, #1705061) was obtained from Biorad. CytoFLEX Sheath Fluid (#B51503) was acquired from Beckman Coulter. Primary antibody for immunofluorescence, p62/SQSTM1 (#NBP1-48320SS) was acquired from Novas Biologicals and Anti-Rabbit IgG Alexa fluor 555 (#A32732) and Anti-Mouse IgG Alexa fluor 488 (#A32723) were procured from Invitrogen. Primary antibodies anti-SQSTM1/p62 (D1Q5S) #39749S, anti-LC3 A/B (D3U4C) #12741S, anti-β-Actin (8H10D10) #3700S, anti-β-tubulin (D3U1W) #86298T, anti-LAMP-1 (D2D11, #9091S) and secondary antibodies viz. anti-mouse (#7076S) and anti-rabbit (#7074P2) were purchased from Cell Signaling Technology (CST). Anti-P-Selectin (CTB201, #sc-8419) was acquired from SantaCruz Biotechnology.

### Animal experiment details

Wistar rats, 6–8 weeks aged with body weight in the range of 120–160 g were obtained from Central Animal Facility at BITS Pilani according to the approved protocol no. IAEC/RES/27/01. Animals were housed in polypropylene cages with temperature maintained at 24 °C and they were fed ad libitum. Experiments performed in this article were approved by Institutional Animal Ethics Committee (IAEC), BITS Pilani (Reg. No.417/PO/ReBi/2001/CPCSEA) and were performed in accordance to IAEC and ARRIVE guidelines and regulations. The animals were anesthetized using isoflurane for blood collection. For *ex-vivo* experiments, blood obtained from individual rats was processed to isolate platelets and then divided into different treatment groups used in this study. For *in-vivo* experiments, individual rats were exposed to different conditions and there after platelets representing respective conditions were isolated and directly used for analysis without *ex-vivo* culture. Further, animals were euthanized by cervical dislocation post inferior vena cava (IVC) ligation and hypoxia exposure for thrombus and organ extraction to minimize their contamination by chemical agents as mentioned in American Veterinary Medical Association (AVMA) guidelines for the euthanasia of animals (2020).

### Isolation of platelet from whole blood

Rats were anaesthetized and blood was collected in acid-citrate-dextrose (ACD) buffer containing eppendorf by retro orbital puncture. Collected blood was distributed in fresh 1.5 ml eppendorf and equal volume of modified Tyrode-HEPES buffer was added to it. The contents were centrifuged for 15 min at 200 g for platelet rich plasma (PRP) separation without brake, as that might lead to activation of platelets. The separated PRP was incubated at room temperature for 5 min. PRP obtained was transferred to 1.5 ml eppendorf along with the treatment and incubated for 10 min. These tubes were re-centrifuged at 800×*g* for 10 min at 22 °C. Obtained pellets were carefully resuspended in modified Tyrode- HEPES buffer.

### Ex-vivo exposure of platelets to hypoxia

The culture dishes were precoated with 1% gelatin for adherence of platelets. Platelets re-suspended in modified Tyrode-HEPES buffer were added to these dishes and kept in hypoxia incubator procured from Thermo Fisher Company (Model 4131) through project sponsored by LSRB, Deference Research and Development Organization for subjecting to variable oxygen concentrations for 30 min. The normoxic conditions were maintained at 21% and hypoxic conditions maintained at 10%^15,40^. Platelets were visualized under inverted microscope before further experimentation. The condition of 10% oxygen concentration was decided based on the article of Peacock et al. which suggests that oxygen pressure fall roughly linearly with altitude and is around 50% of the sea level (21% oxygen) at high altitudes. Therefore, 10% oxygen concentration was selected for the study mimicking high altitude associated hypoxia.

For *ex-vivo* experiments, platelets were exposed to 30 min of hypoxia; long term *ex-vivo* hypoxic exposure resulted in increased clumping of platelets.

### Microscopic imaging of isolated and cultured platelets

Upon adhesion, platelets were visualized and imaged under microscope post 30 min exposure to 21% (normoxic) and 10% (hypoxic) oxygen concentrations using Zeiss Axiocam 105 color microscope. Following this 4% paraformaldehyde treatment was given to the platelets for 10–15 min for fixation and phalloidin (1:1000) and DAPI (1:1000) stains were added thereafter for platelet characterization.

### Platelet aggregation

Isolated and treated platelets were seeded in 96-well plate and kept under varying oxygen conditions inside the incubator for 30 min. Post this, readings were taken at 405 nm for 10 min with 2 min shaking at high speed in the middle using Multiskan sky spectrophotometer procured from Thermoscientific and light absorbance was measured^7,17^.

### Scanning electron microscopy

Culture dishes were coated with 1% gelatin for 1 h and isolated platelets were subjected to variable oxygen concentrations (21% and 10% O_2_). Post 30 min, platelets were treated with 4% paraformaldehyde for fixation followed by PBS wash. The samples were dehydrated chronologically using 50%, 75% and 100% ethanol for 1 min each and were then air dried. Samples were coated with thin film of chromium in a vacuum coater before visualization using Apreo S of FEI (Thermo Fisher) field emission scanning electron microscopy (FESEM).

### Cytotoxicity analysis

Platelets were cultured in gelatin coated 96-well plates along with specific treatments for 30 min. MTT assay was used to determine cytotoxicity wherein live upon its addition, live cells form formazan crystals which are further dissolved in DMSO, following which absorbance was measured at 570 nm and 630 nm using Multiskan sky spectrophotometer (Thermo Fisher). Normoxia and hypoxia exposed platelets without any other treatment served as control for calculating relative percentage cellular viability.

### Static adhesion and platelet spreading assay

18 mm coverslips were coated with collagen (20ug/ml) and kept at 37℃ as per manufacturer’s protocol. Isolated platelets were distributed on these collagen-coated cover slips and post 30 min of hypoxia exposure paraformaldehyde was added to fix the cells. After Triton-X treatment, platelets were stained with phalloidin (1:1000) for 30 min and mounted using glycerol. These slides were further viewed under microscope (Zeiss)^19^.

### Clot retraction assay

PRP was treated with chloroquine for 10 min and 1U thrombin (platelet activation inducer) was added followed by immediately stirring and sealing the test tube^41^. They were then exposed to hypoxic and normoxic conditions for 30 min and retraction of clot was analyzed.

### Lysotracker staining

Washed platelets obtained from whole blood were exposed to 21% (normoxic) and 10% (hypoxic) conditions. 1 μM lysotracker dilution was added to the eppendorfs and an incubation of 15-20 min was given to the samples at 37℃, followed by centrifugation at 400×*g* for 5 min and resuspension of pellets in buffer. Sample acquisition was done using flow cytometer (Cytoflex S, Beckmann Coulter) followed by data analysis by CytExpert 2.4 software.

### Immunofluorescence

Platelets exposed to variable oxygen conditions for 30 min. 4% paraformaldehyde was used for fixation and 0.1% Triton-X was added for permeabilization. 2.5% BSA solution was used for blocking so as to avoid any kind of non-specific interaction. After overnight primary antibody incubation, secondary antibody was added and incubated for 2 h post which phalloidin staining was done. The slides were carefully mounted and imaged using Zeiss LSM880 confocal microscopy. Additional details of microscope settings are provided in the ‘Equipment and settings’ section.

### Flow cytometry

Washed platelets were incubated under 10% O_2_ conditions for 30 min and primary antibody P-Selectin (1:500) was directly added inside the incubator to avoid re-oxygenation. After 30 min incubation with primary antibody, platelets were centrifuged at 400xg for 5 min and then Alexa fluor 488 (1:1000) was added to the obtained pellets. It was again incubated for 30 min and further centrifuged. The obtained pellets were carefully re-suspended in flow buffer, following which samples were acquired by flow cytometer (Cytoflex S, Beckmann Coulter) and data was analyzed using CytExpert 2.4.

### Immunoblotting

A cocktail containing platelet lysis buffer (2% NP40, 150 mM NaCl, 30 mM HEPES, 2 mM EDTA) and PMSF (Sigma Aldrich) was added to the samples post 30 min hypoxia exposure inside the incubator to avoid re-oxygenation. They were kept in 4℃ for 30 min and then stored in -20℃ before further processing. Samples were snap thawed and pipetted vigorously for cell membrane lysis. After centrifugation at 12000 rpm for 20 min at 4℃, the supernatant was collected. Bradford assay was done to estimate the amount of obtained protein. For sample preparation, 1X buffer was added to loading amount of protein and heat denatured in dry bath at 100℃ for 10 min. Final samples were loaded in the wells created on polyacrylamide gel and protein separation was carried out with SDS-PAGE technique. The contents of gel were relocated to PVDF membrane using semi dry transfer apparatus (Biorad) followed by blocking of non-specific sites using 5% skimmed milk in TBS. The membranes were probed and re-probed with primary antibody (1:750 dilution) overnight at 4℃ and were subjected to secondary antibody (1:1000 dilution) incubation for 2 h at room temperature. Horseradish peroxide-conjugated goat antirabbit IgG were used as secondary antibodies. Post washing of any excess antibody from the membranes, enhanced chemiluminescence detection system (Biorad, Molecular Imager ChemiDoc XRS + with Image Lab Software) was used for spotting protein intensities. Densitometric quantification of the detected proteins was performed by ImageJ 1.53a software by normalization using control.

### Animal exposure to hypoxic conditions

Animals were treated with chloroquine (5 mg/kg) 30-45 min before exposure to hypoxic or normoxic conditions. They were kept in a sealed chamber under experimental conditions for 24 h with a gas exchange and refill of 5-10 min after every 3 h to avoid CO_2_ saturation. Long-term hypoxia exposure beyond 24 h resulted in weakness and health deterioration of the animals and hence were not considered for the current study. Blood collected from these animals was used to study parameters related to platelet aggregation.

### Tail bleeding and blood volume analysis

Animals were anaesthetized and a cut was made at 3-5 mm distance from the tip of its tail. Blood was collected in an eppendorf containing PBS and time taken for cessation of bleeding was noted^42^. Volume of collected blood was subsequently measured.

### Flow restriction animal model

Animals were given ketamine (40 mg/kg) and xylazine (10 mg/kg) according to their body weights. A blood flow restriction model was developed to initiate thrombus formation by proximal ligation of inferior vena cava (IVC) and its lateral tributaries. The formed thrombus was collected after 24 h of surgery and measured to compare the effects of treatment with that of control^30^. Post surgery the animals were given gentamycin (25 mg/kg) and celecoxib (10 mg/kg) treatment in order to avoid infection and pain.

### Histology

Organ samples and thrombus were stored in formalin after extraction for fixation and were outsourced to Advanced Histology Labs, New Delhi for slide preparation. Briefly, the process followed for slide preparation consists of block preparation and subsequent sectioning of the samples which were then placed on slides and stained using haematoxylin and eosin stain. The obtained slides were imaged under microscope (Zeiss).

### Mass spectrometry sample preparation and analysis

Whole blood samples were collected from animal post CQ treatment and 24 h of hypoxia exposure. Platelets were isolated and protein was extracted from the samples using lysis buffer. Obtained whole cell lysates were outsourced to Valerian Chem Private Limited, New Delhi for mass spectrometry. Briefly, protein per sample was used for digestion and reduced with 5 mM TCEP. Further 50 mM iodoacetamide treatment was given for alkylation followed by digestion with Trypsin (1:50, Trypsin/lysate ratio) for 16 h at 37 °C. C18 silica cartridge was used to clean the digests by removing salt accompanied by speed vac for drying. The dried pellet was re-suspended in buffer A (2% acetonitrile, 0.1% formic acid). Experiments were performed on an Easy-nlc-1000 system coupled with an Orbitrap Exploris mass spectrometer. 1ug of peptide sample were loaded on C18 column 15 cm, 3.0 μm Acclaim PepMap (Thermo Fisher Scientific) and separated with a 0–40% gradient of buffer B (80% acetonitrile, 0.1%formic acid) at a flow rate of 500 nl/min) and injected for MS analysis. LC gradients were run for 110 min. MS1 spectra were acquired in the Orbitrap (Max IT = 60 ms, AGQ target = 300%; RF Lens = 70%; R = 60 K, mass range = 375 − 1500; Profile data). Dynamic exclusion was employed for 30 s excluding all charge states for a given precursor. MS2 spectra were collected for top 20 peptides. MS2 (Max IT = 60 ms, R = 15 K, AGC target 100%). All samples were processed and RAW files generated were analyzed with Proteome Discoverer (v2.5) against the Uniprot Rattus database. For dual Sequest and Amanda search, the precursor and fragment mass tolerances were set at 10 ppm and 0.02 Da, respectively. The protease used to generate peptides, i.e. enzyme specificity was set for trypsin/P (cleavage at the C terminus of “K/R: unless followed by “P”). Carbamidomethyl on cysteine as fixed modification and oxidation of methionine and N-terminal acetylation were considered as variable modifications for database search. Both peptide spectrum match and protein false discovery rate were set to 0.01 FDR.

### Equipment and settings

The immunoblots were probed with antibodies (procured from CST or Abcam) at specific dilutions as mentioned in the ‘Immunoblotting’ Section. Blot images were captured using gel doc (Biorad, Molecular Imager Chemidoc XRS +), acquired using Biorad Image Lab 6.1 software, and further analyzed using ImageJ 1.53a. For clarity and conciseness of presentation, blots were cropped using Image Lab 6.1 and represented in resolution—600dpi. The original uncropped images, their lane sequence, and molecular size markings are presented in the Supplementary Material as indicated in the respective figure legend. The molecular size (kDa) of each protein analyzed is mentioned in the main figure. Immunofluorescence images were acquired using Zeiss LSM880 AxioObserver microscope; Objective- Plan-Apochromat 63x/1.40 Oil DIC M27; Filters- 566–697, -2147483648–2147483648, 493–558; Beam splitter- MBS:MBS 458/561 (for LAMP1 and p62), or MBS:MBS 488 (for Phalloidin) and MBS_InVis : Plate; Mirror- DBS1. Laser was adjusted according to the excitation and emission wavelengths with intensity percentage ranging from 2.8 to 5.5%. The other microscope settings were as follows—(A) For red channel, the excitation wavelength was 561 nm and emission wavelength was 632 nm. (B) For green channel, the excitation wavelength was 488 nm and emission wavelength was 526 nm. Images were processed with ZEN 3.2 (ZEN lite) software where gamma changes were set between 0.7–1.4. Settings were applied equally across individual experimental panel. For flow cytometry experiments the data were captured using Cytoflex S (Beckmann Coulter) and analyzed using CytExpert 2.4 software. For scanning electron microscopy, the images were acquired under high vacuum mode using Apreo S of FEI (Thermo Fisher) field emission scanning electron microscopy (FESEM) at magnification specified in the respective figure legends.

### Statistical analysis

Obtained data was analyzed using GraphPad Prism software version 8.0.1 and plotted applying mean with SD. Statistical determination of role of hypoxia with and without treatment in contrast to control was determined using unpaired t-test. Multiple comparisons were examined using Turkey method with significance defined as p-value ≤ 0.05.

## Supplementary Information

Supplementary Information 1.

Supplementary Information 2.

Supplementary Information 3.

## Data Availability

“Data is provided within the manuscript or supplementary information files”.

## References

[1] Brewer, D. B. Max Schultze (1865), G. Bizzozero (1882) and the discovery of the platelet. *Br. J. Haematol.* **133**(3), 251–258. 10.1111/j.1365-2141.2006.06036.x (2006). 16643426 10.1111/j.1365-2141.2006.06036.x

[2] Linden, M. D. *Platelet Physiology* 992 (Humana Press, 2013).

[3] van der Meijden, P. E. J. & Heemskerk, J. W. M. Platelet biology and functions: New concepts and clinical perspectives. *Nat. Rev. Cardiol.* **16**(3), 166–179. 10.1038/s41569-018-0110-0 (2019). 30429532 10.1038/s41569-018-0110-0

[4] Twomey, L. et al. Platelets: From formation to function. *Homeostasis*10.5772/intechopen.80924 (2019).

[5] Shin, E. K., Park, H., Noh, J. Y., Lim, K. M. & Chung, J. H. Platelet shape changes and cytoskeleton dynamics as novel therapeutic targets for anti-thrombotic drugs. *Biomol. Ther.* **25**(3), 223–230. 10.4062/biomolther.2016.138 (2017). 10.4062/biomolther.2016.138 PMC5424631 27871158

[6] Yun, S. H., Sim, E. H., Goh, R. Y., Park, J. I. & Han, J. Y. Platelet activation: The mechanisms and potential biomarkers. *Biomed. Res. Int.* **2016**, 10–15. 10.1155/2016/9060143 (2016). 10.1155/2016/9060143 PMC4925965 27403440

[7] Ghoshal, K. & Bhattacharyya, M. Overview of platelet physiology: Its hemostatic and nonhemostatic role in disease pathogenesis. *Sci. World J.* **2014**, 1–17. 10.1155/2014/781857 (2024). 10.1155/2014/781857 PMC3960550 24729754

[8] Ouseph, M. M. et al. Autophagy is induced upon platelet activation and is essential for hemostasis and thrombosis. *Blood* **126**(10), 1224–1233. 10.1182/blood-2014-09-598722 (2015). 26209658 10.1182/blood-2014-09-598722 PMC4559933

[9] Mizushima, N. Autophagy: Process and function. *Genes Dev.* **21**(22), 2861–2873. 10.1101/gad.1599207 (2007). 18006683 10.1101/gad.1599207

[10] Badadani, M. Autophagy mechanism, regulation, functions, and disorders. *ISRN Cell Biol.* **2012**(2), 1–11. 10.5402/2012/927064 (2012).

[11] Chen, P. S. et al. Pathophysiological implications of hypoxia in human diseases. *J. Biomed. Sci.* **27**(1), 1–19. 10.1186/s12929-020-00658-7 (2020). 32389123 10.1186/s12929-020-00658-7 PMC7212687

[12] Gupta, N., Zhao, Y. Y. & Evans, C. E. The stimulation of thrombosis by hypoxia. *Thromb Res.* **181**, 77–83. 10.1016/j.thromres.2019.07.013 (2019). 31376606 10.1016/j.thromres.2019.07.013

[13] Ninivaggi, M. et al. Hypoxia induces a prothrombotic state independently of the physical activity. *PLoS ONE* **10**(10), 141797. 10.1371/journal.pone.0141797 (2015). 10.1371/journal.pone.0141797 PMC4627841 26516774

[14] Yan, S. F., Mackman, N., Kisiel, W., Stern, D. M. & Pinsky, D. J. Hypoxia/hypoxemia-induced activation of the procoagulant pathways and the pathogenesis of ischemia-associated thrombosis. *Arterioscler. Thromb. Vasc. Biol.* **19**(9), 2029–2035. 10.1161/01.ATV.19.9.2029 (1999). 10479642 10.1161/01.atv.19.9.2029

[15] Peacock, A. ABC of oxygen. Oxygen at high altitude. *Br. Med. J.* **317**(7165), 1063–1066. 10.1136/bmj.317.7165.1063 (1998). 9774298 10.1136/bmj.317.7165.1063 PMC1114067

[16] Report, C. Pulmonary embolism in young natives of high altitude. *Heart Views* **17**(2), 62–65. 10.4103/1995-705X.185115 (1995). 10.4103/1995-705X.185115 PMC4966210 27512534

[17] Chan, M. V., Armstrong, P. C. & Warner, T. D. 96-well plate-based aggregometry. *Platelets* **29**(7), 650–655. 10.1080/09537104.2018.1445838 (2018). 29543546 10.1080/09537104.2018.1445838 PMC6178088

[18] Brain, E. & Du, X. New concepts and mechanisms of platelet activation signaling. *Physiology* **32**, 162–177. 10.1152/physiol.00020.2016 (2017). 28228483 10.1152/physiol.00020.2016 PMC5337829

[19] Kulkarni, P. P., Sonkar, V. K., Gautam, D. & Dash, D. AMPK inhibition protects against arterial thrombosis while sparing hemostasis through differential modulation of platelet responses. *Thromb. Res.* **196**(March), 175–185. 10.1016/j.thromres.2020.08.033 (2020). 32890901 10.1016/j.thromres.2020.08.033

[20] Tucker, K. L., Sage, T. & Gibbins, J. M. Europe PMC Funders Group Clot retraction. *Methods Mol. Biol.* **4**, 101–107. 10.1007/978-1-61779-307-3 (2019). 10.1007/978-1-61779-307-3_8 PMC6446996 22130703

[21] Lee, T. Y. et al. Platelet autophagic machinery involved in thrombosis through a novel linkage of AMPK-MTOR to sphingolipid metabolism. *Autophagy* **17**(12), 4141–4158. 10.1080/15548627.2021.1904495 (2021). 33749503 10.1080/15548627.2021.1904495 PMC8726689

[22] Cheng, X. et al. Revisiting LAMP1 as a marker for degradative autophagy-lysosomal organelles in the nervous system. *Autophagy* **14**(8), 1472–1474. 10.1080/15548627.2018.1482147 (2018). 29940787 10.1080/15548627.2018.1482147 PMC6103665

[23] Cui, L. et al. The lysosomal membrane protein lamp2 alleviates lysosomal cell death by promoting autophagic flux in ischemic cardiomyocytes. *Front. Cell Dev. Biol.* **8**, 1–14. 10.3389/fcell.2020.00031 (2020). 32117965 10.3389/fcell.2020.00031 PMC7019187

[24] Monaci, S. et al. Hypoxia induces autophagy in human dendritic cells: Involvement of class III PI3K / Vps34. *Cells* **11**(10), 1–16. 10.3390/cells11101695 (2022). 10.3390/cells11101695 PMC9139568 35626732

[25] Murugan, S. & Amaravadi, R. K. *Methods for Studying Autophagy Within the Tumor Microenvironment* 145–166 (Springer, 2016). 10.1007/978-3-319-26666-4_9 PMC5451257 27325266

[26] Mauthe, M. et al. Chloroquine inhibits autophagic flux by decreasing autophagosome-lysosome fusion. *Autophagy* **14**(8), 1435–1455. 10.1080/15548627.2018.1474314 (2018). 29940786 10.1080/15548627.2018.1474314 PMC6103682

[27] Halcrow, P. W., Geiger, J. D. & Chen, X. Overcoming chemoresistance: Altering pH of Cellular compartments by chloroquine and hydroxychloroquine. *Front Cell Dev. Biol.* **9**(February), 1–14. 10.3389/fcell.2021.627639 (2021). 10.3389/fcell.2021.627639 PMC7900406 33634129

[28] Bearer, E. L., Prakash, J. M. & Li, Z. *Actin Dynamics in Platelets* 217 (Elsevier, 2002). 10.1016/s0074-7696(02)17014-8 PMC3376087 12019562

[29] Agbani, E. O. et al. Carbonic anhydrase inhibitors suppress platelet procoagulant responses and in vivo thrombosis. *Platelets* **00**(00), 1–7. 10.1080/09537104.2019.1709632 (2020). 10.1080/09537104.2019.1709632 31893963

[30] Tyagi, T. et al. Altered expression of platelet proteins and calpain activity mediate hypoxia-induced prothrombotic phenotype. *Blood* **123**(8), 1250–1260. 10.1182/blood-2013-05-501924 (2014). 24297866 10.1182/blood-2013-05-501924

[31] Delaney, C. et al. Platelet activation contributes to hypoxia-induced inflammation. *Am. J. Physiol.*10.1152/ajplung.00519.2020 (2020). 10.1152/ajplung.00519.2020 PMC8294621 33264579

[32] Jones, C. I. et al. Integrin-linked kinase regulates the rate of platelet activation and is essential for the formation of stable thrombi. *J. Thromb. Haemost.* **12**(8), 1342–1352. 10.1111/jth.12620 (2014). 24888521 10.1111/jth.12620

[33] Nelson, C. M. Soft microenvironments induce chemoresistance by increasing autophagy downstream of integrin-linked kinase. *Cancer Res.* **80**(2), 4103–4113. 10.1158/0008-5472.CAN-19-4021 (2020). 33008805 10.1158/0008-5472.CAN-19-4021 PMC7534696

[34] Zhou, J., May, L., Liao, P., Gross, P. L. & Weitz, J. I. Expression and venous thrombosis in rats. *ATVB* **29**, 863–869. 10.1161/ATVBAHA.109.185678 (2009). 10.1161/ATVBAHA.109.185678 19265029

[35] Feng, W. et al. Dissection of autophagy in human platelets. *Autophagy* **10**(4), 642–651. 10.4161/auto.27832 (2014). 24458007 10.4161/auto.27832 PMC4091151

[36] You, T., Wang, Q. & Zhu, L. Role of autophagy in megakaryocyte differentiation and platelet formation. *Int. J. Physiol. Pathophysiol. Pharmacol.* **8**(1), 28–34 (2016). 27186320 PMC4859876

[37] Sun, R. J. & Shan, N. N. Megakaryocytic dysfunction in immune thrombocytopenia is linked to autophagy. *Cancer Cell Int.* **19**(1), 1–10. 10.1186/s12935-019-0779-0 (2019). 30923461 10.1186/s12935-019-0779-0 PMC6419848

[38] Cao, Y. et al. Loss of autophagy leads to failure in megakaryopoiesis, megakaryocyte differentiation, and thrombopoiesis in mice. *Exp. Hematol.* **43**(6), 488–494. 10.1016/j.exphem.2015.01.001 (2015). 25591498 10.1016/j.exphem.2015.01.001

[39] Tang, H., Gao, M., Fu, Y., Gui, R. & Ma, X. The effect of autophagic activity on the function of apheresis platelets and on the efficacy of clinical platelet transfusion. *Transfus. Med. Hemother.* **47**(4), 302–313. 10.1159/000504764 (2020). 32884503 10.1159/000504764 PMC7443674

[40] Jindal, A. K. The highest battlefield of the world: Medical problems and solutions. *Med. J. Armed Forces India* **65**(2), 170–172. 10.1016/S0377-1237(09)80135-4 (2009). 27408227 10.1016/S0377-1237(09)80135-4 PMC4921426

[41] Brass, L. F. Thrombin and platelet activation. *Chest* **124**(3 SUPPL.), 18S-25S. 10.1378/chest.124.3_suppl.18S (2003). 12970120 10.1378/chest.124.3_suppl.18s

[42] Lieschke, F., Zheng, Y., Schaefer, J. H., van Leyen, K. & Foerch, C. Measurement of platelet function in an experimental stroke model with aspirin and clopidogrel treatment. *Front. Neurol.* **11**(February), 1–9. 10.3389/fneur.2020.00085 (2020). 32117036 10.3389/fneur.2020.00085 PMC7026492

