# Supplementary material for: Inhibition of autophagy in platelets as a therapeutic strategy preventing hypoxia induced thrombosis
Source: Sci Rep. 2025 Feb 26;15:6855. doi: 10.1038/s41598-025-91181-y (PMC11865581; doi:10.1038/s41598-025-91181-y)

Replicates of Immunoblots

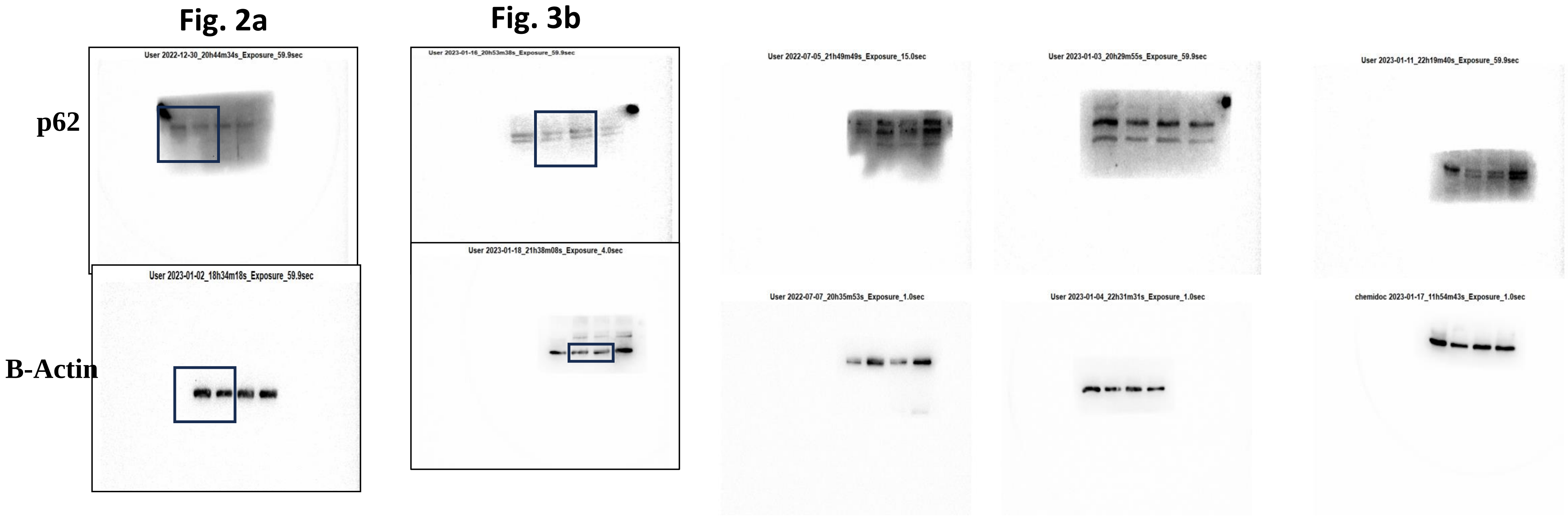

Sequence (from left to right): Lane 1 – C, Lane 2 – H, Lane 3 – H+CQ, Lane 4 – CQ

Key: C – Control (Normoxia; 21% Oxygen),
H – Hypoxia (10% Oxygen),
H+CQ – Hypoxia+Chloroquine (10% Oxygen+2µM)
CQ – Chloroquine (21% Oxygen+ 2µM)

Replicates of Immunoblots

Fig. 2a

Fig. 3b

LC3

B-Actin

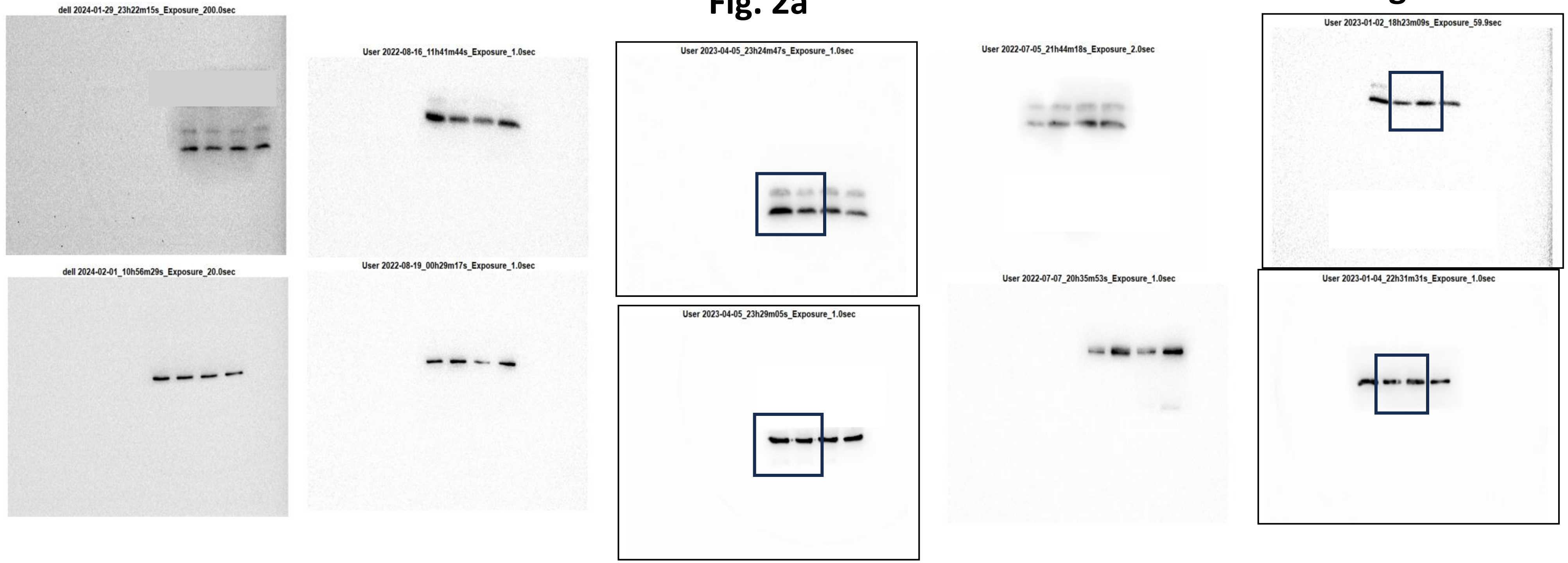

Sequence (from left to right): Lane 1 – C, Lane 2 – H, Lane 3 – H+CQ, Lane 4 – CQ

Key: C – Control (Normoxia; 21% Oxygen),
H – Hypoxia (10% Oxygen),
H+CQ – Hypoxia+Chloroquine (10% Oxygen+2µM)
CQ – Chloroquine (21% Oxygen+ 2µM)

Replicates of Immunoblots

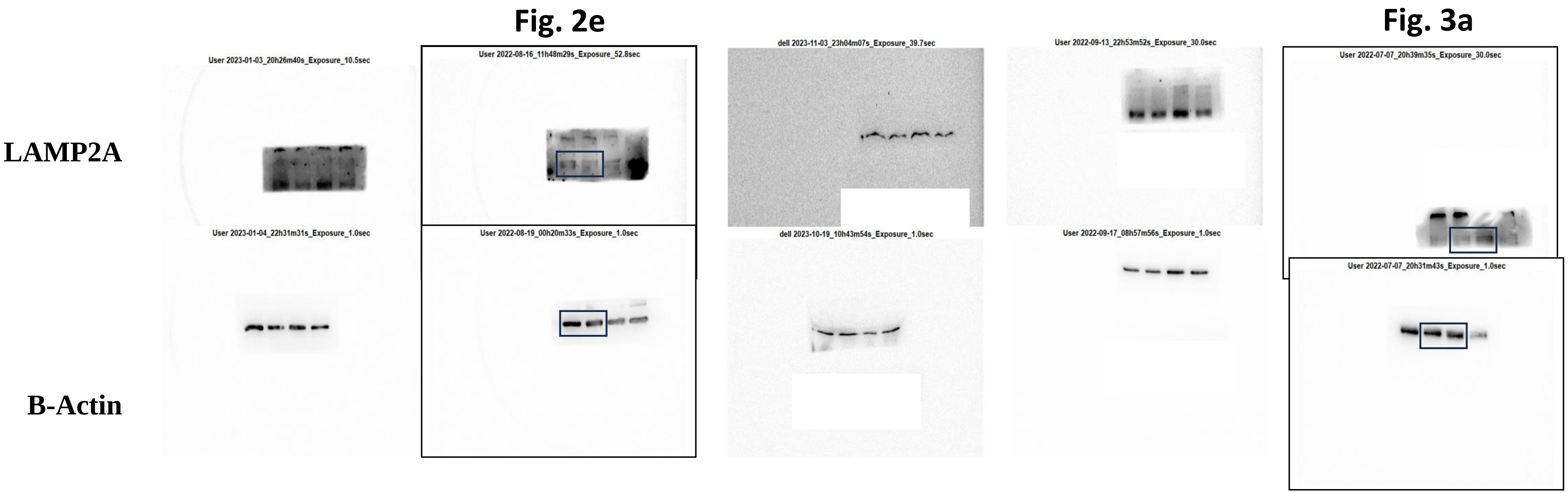

Sequence (from left to right): Lane 1 – C, Lane 2 – H, Lane 3 – H+CQ, Lane 4 – CQ

Key: C – Control (Normoxia; 21% Oxygen),
H – Hypoxia (10% Oxygen),
H+CQ – Hypoxia+Chloroquine (10% Oxygen+2µM)
CQ – Chloroquine (21% Oxygen+ 2µM)

Replicates of Immunoblots *(In-vivo)*

Fig. 5e

p62

B-Tubulin

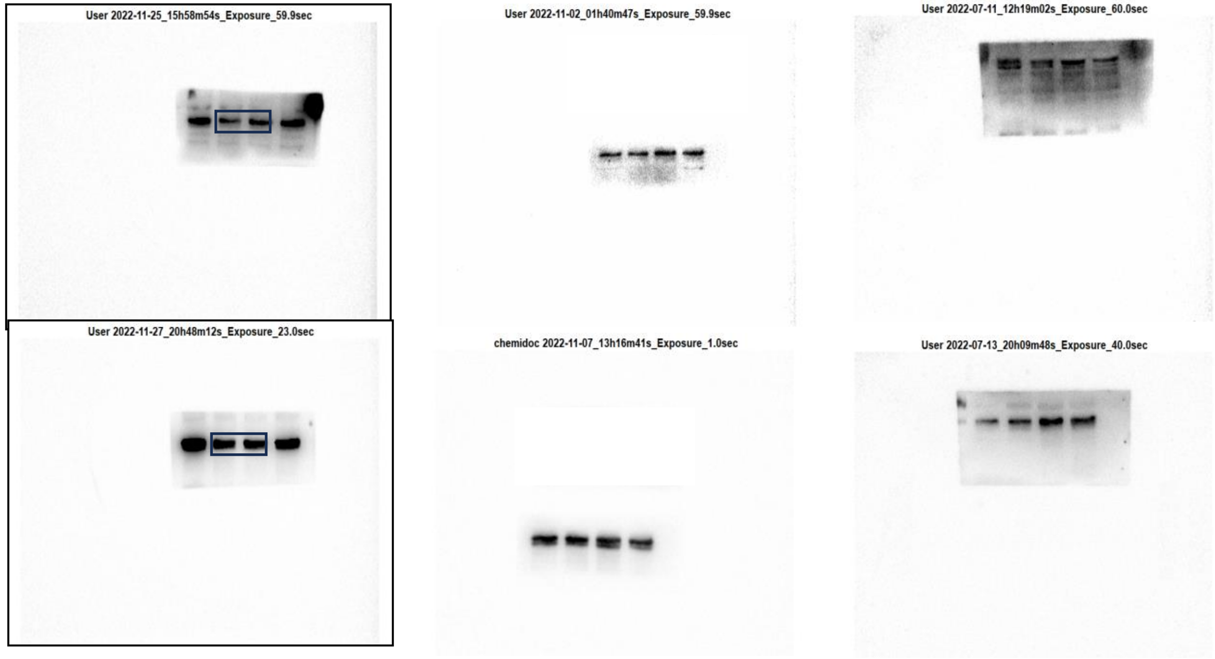

Sequence (from left to right): Lane 1 – C, Lane 2 – H, Lane 3 – H+CQ, Lane 4 – CQ

Key: C – Control (Normoxia; 21% Oxygen),
H – Hypoxia (10% Oxygen),
H+CQ – Hypoxia+Chloroquine (10% Oxygen+5mg/Kg)
CQ – Chloroquine (21% Oxygen+5mg/Kg)

Replicates of Immunoblots *(In-vivo)*

Fig. 5e

LC3

B-Tubulin

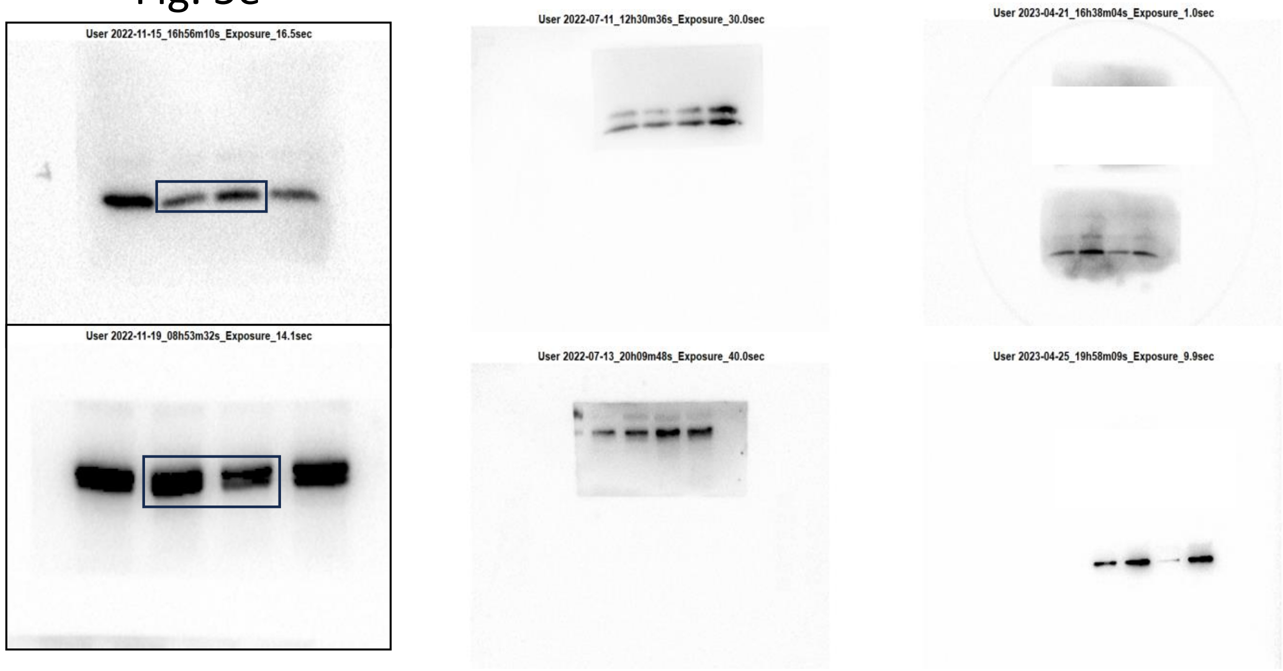

Sequence (from left to right): Lane 1 – C, Lane 2 – H, Lane 3 – H+CQ, Lane 4 – CQ

Key: C – Control (Normoxia; 21% Oxygen),
H – Hypoxia (10% Oxygen),
H+CQ – Hypoxia+Chloroquine (10% Oxygen+5mg/Kg)
CQ – Chloroquine (21% Oxygen+5mg/Kg)

Immunofluorescence (p62)

Cntrl

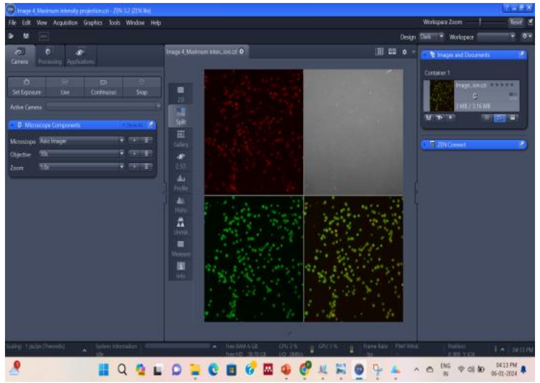

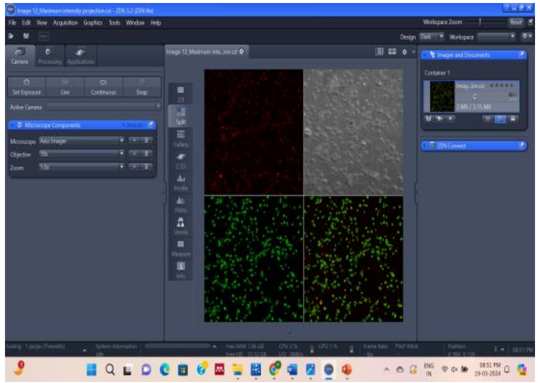

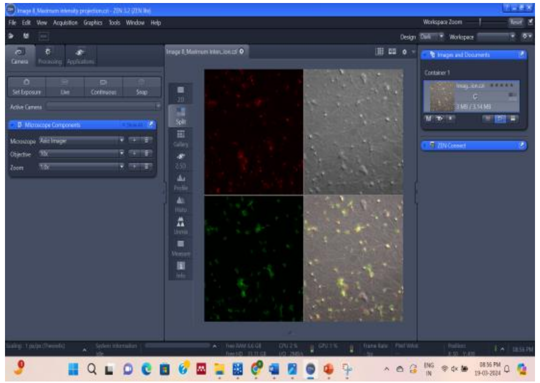

Hyp

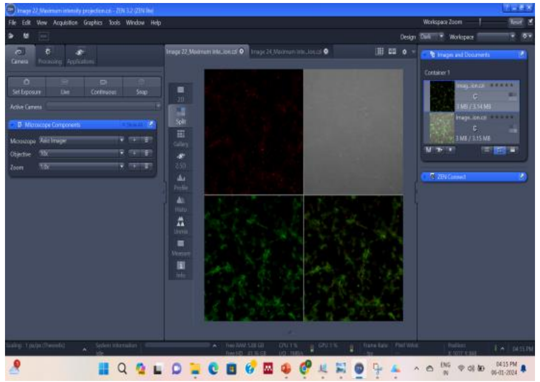

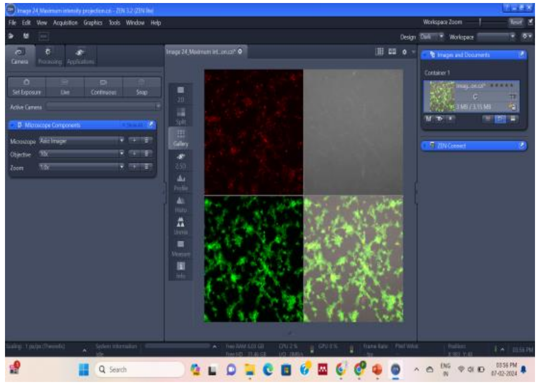

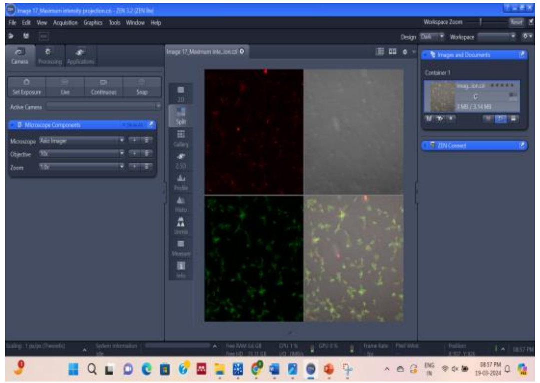

Hyp+CQ

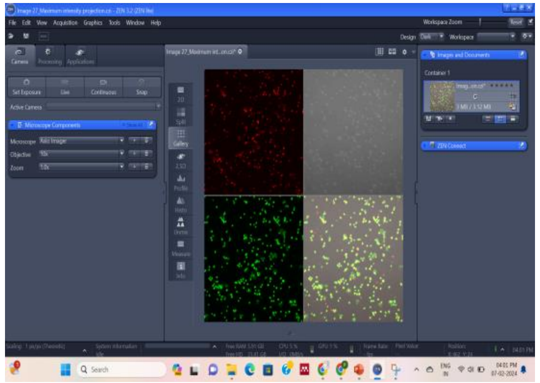

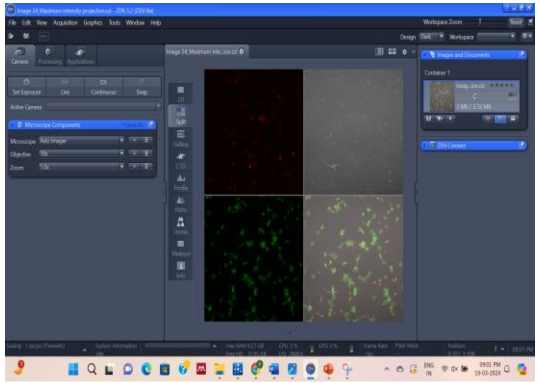

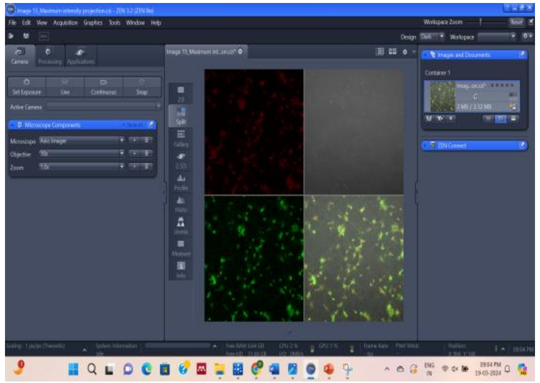

Immunofluorescence (LAMP1)

Cntrl

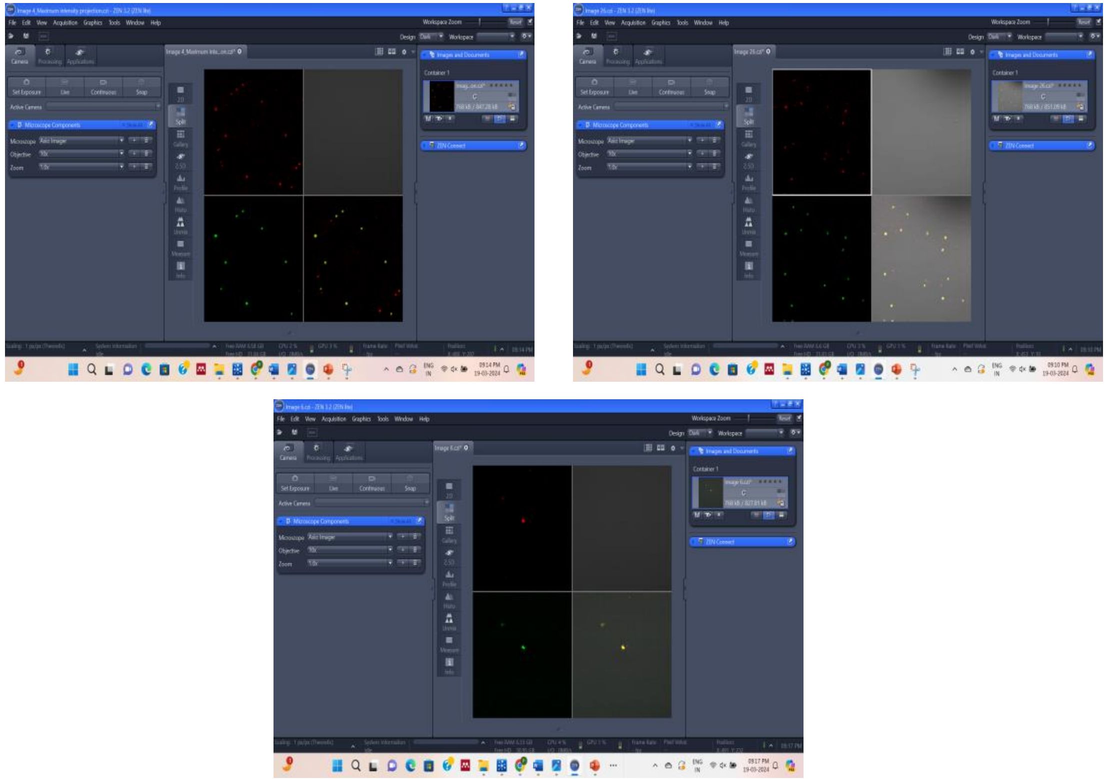

Hyp

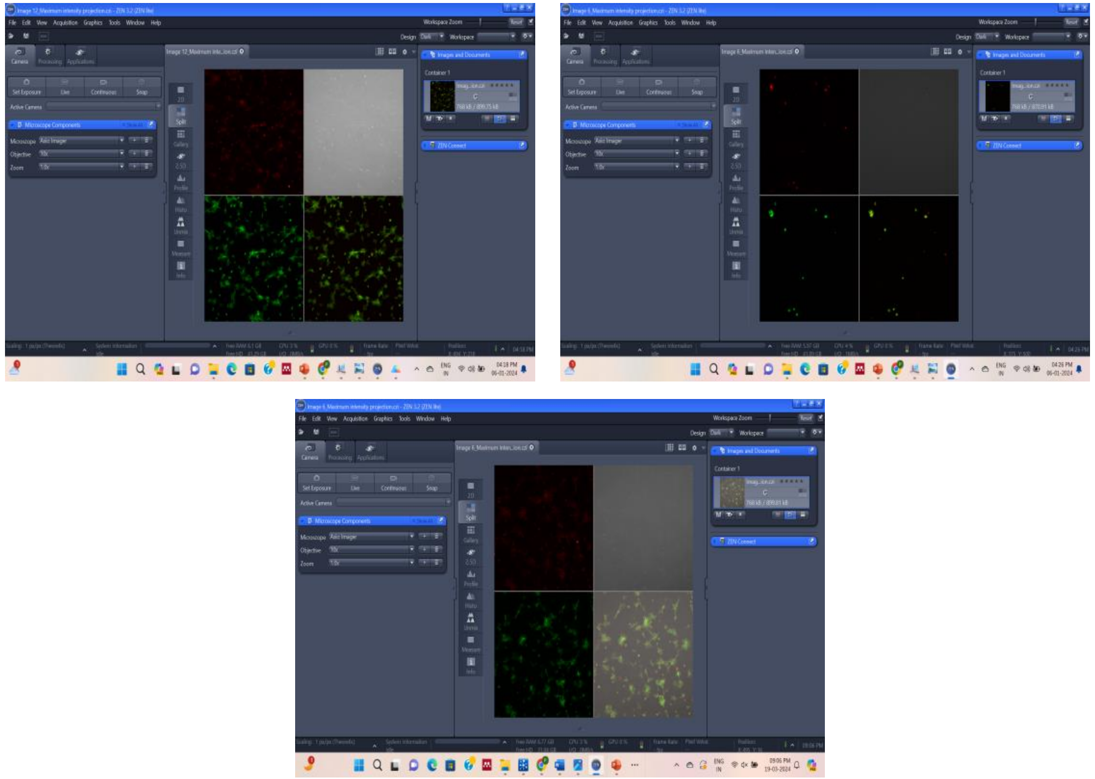

Hyp+CQ

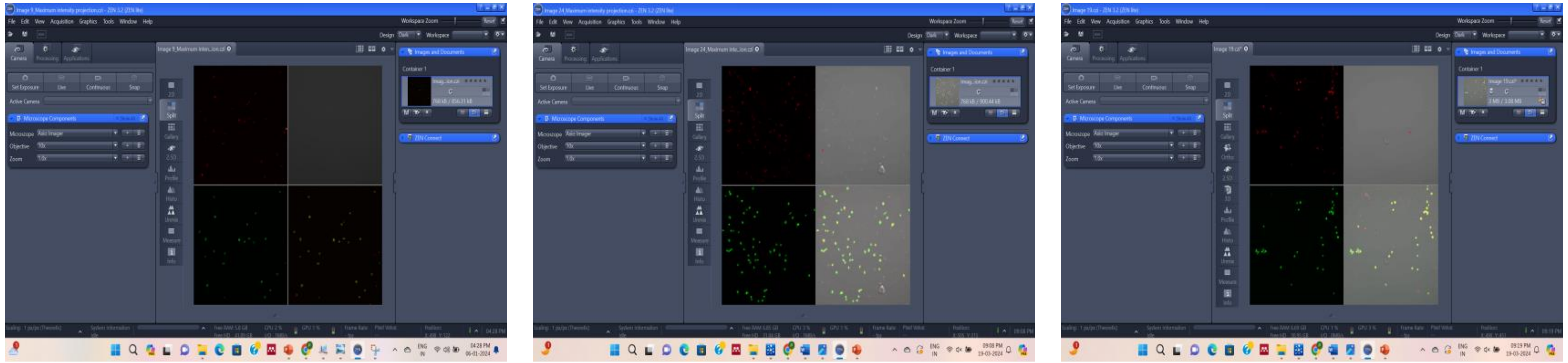

Phalloidin

Cntrl

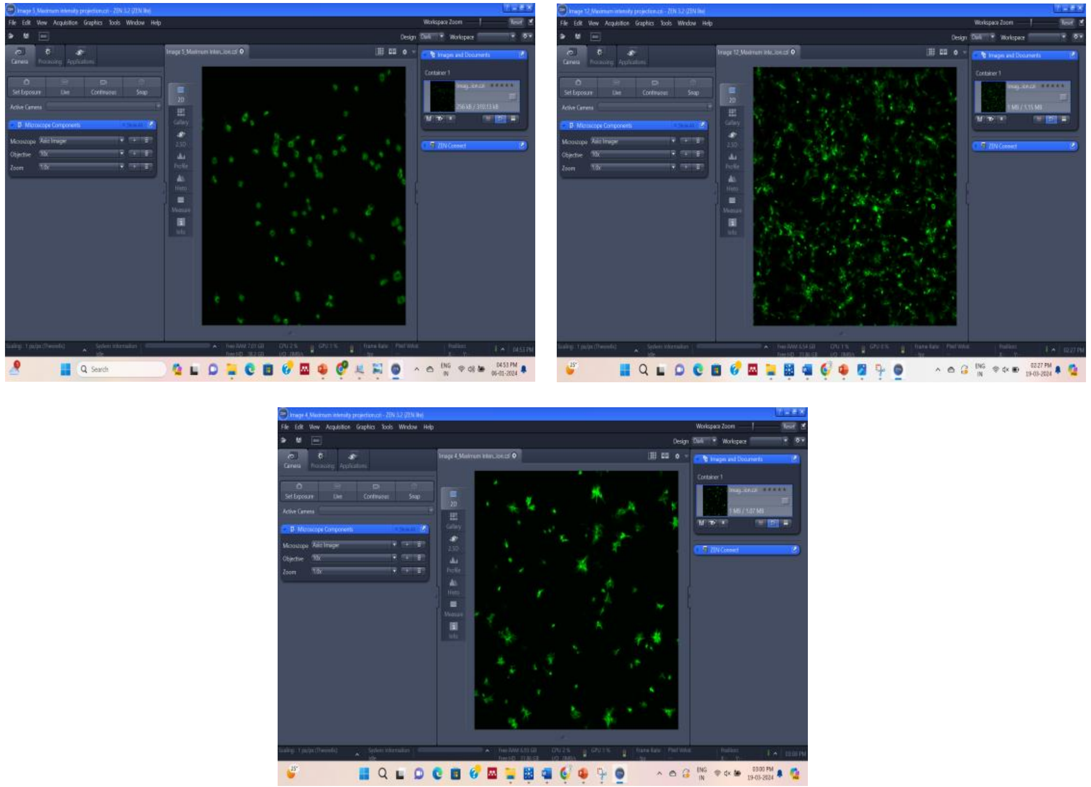

Hyp

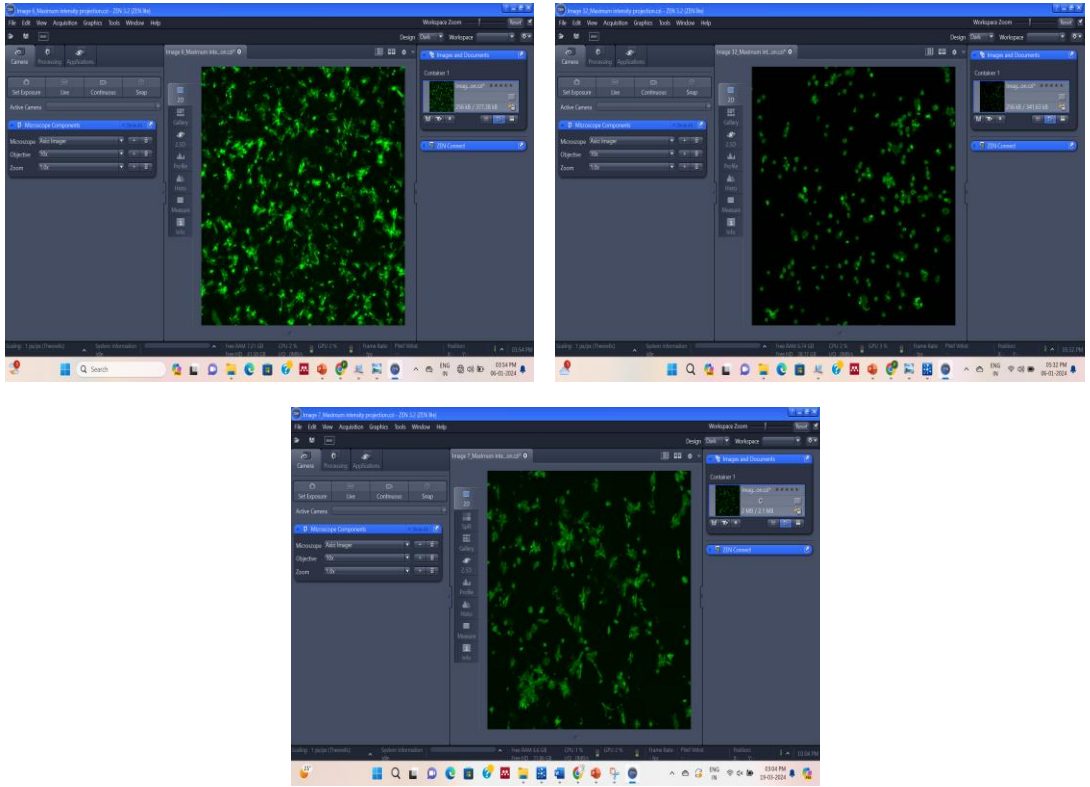

Hyp+CQ

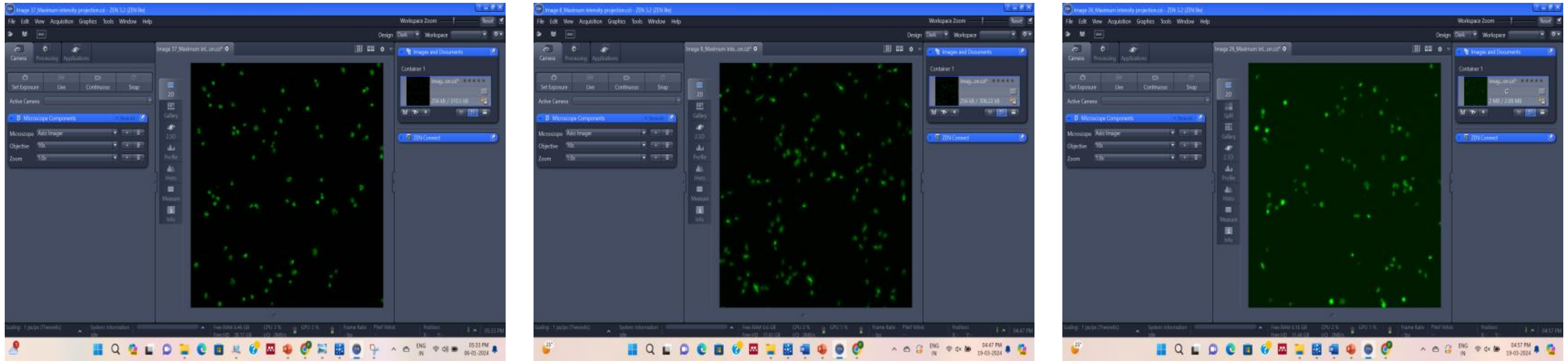

SEM images

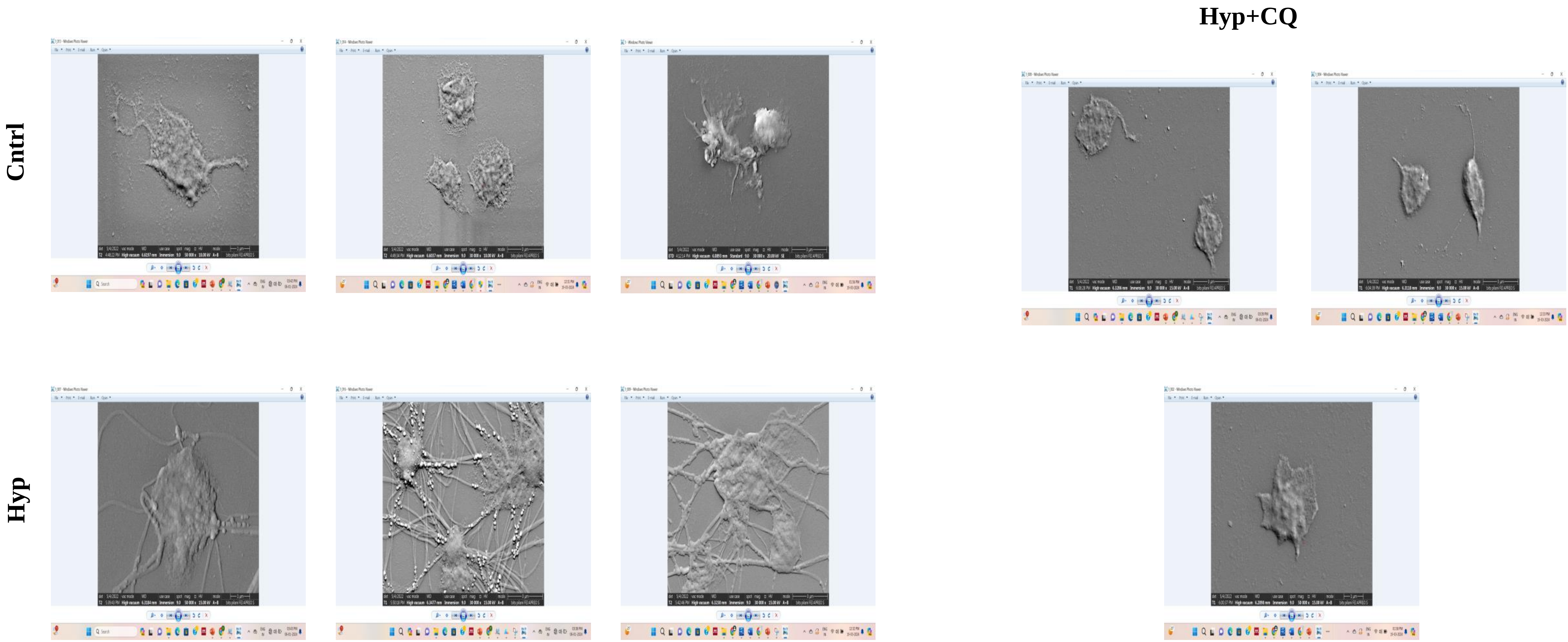

Phase-contrast of Platelet

Hyp+CQ

Cntrl

Hyp

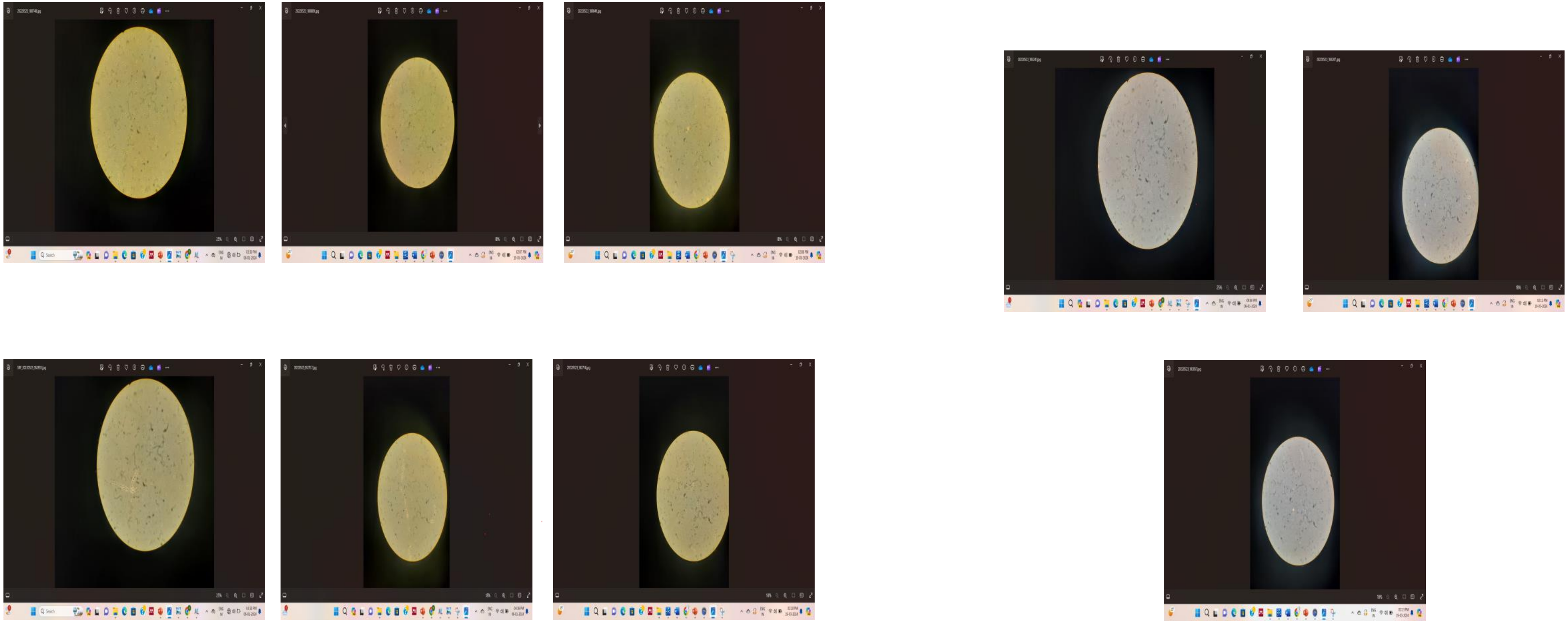

Flow Cytometry

Lysotracker (*Ex-Vivo*)

Cntrl

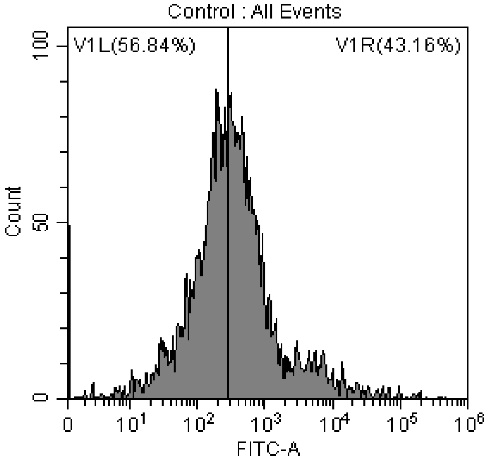

Hyp

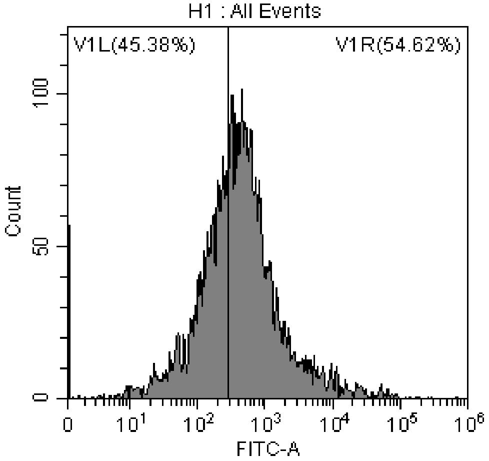

Cntrl

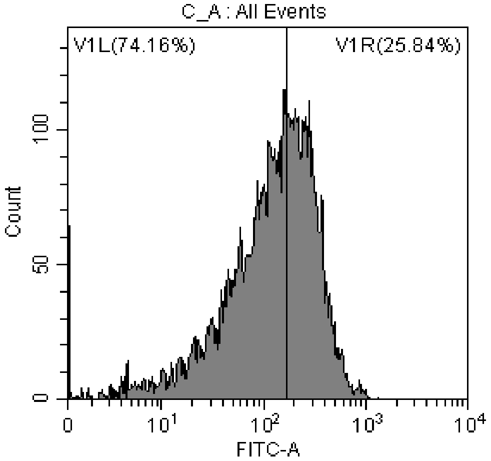

Hyp

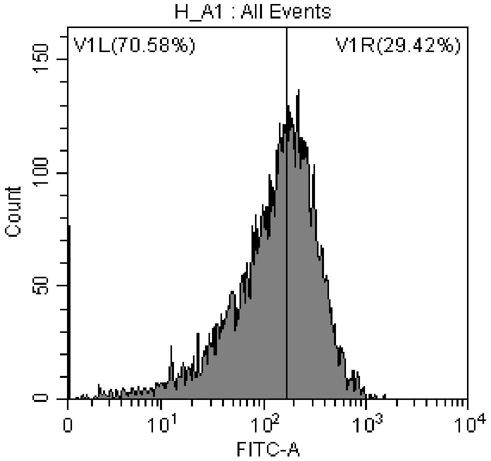

Cntrl

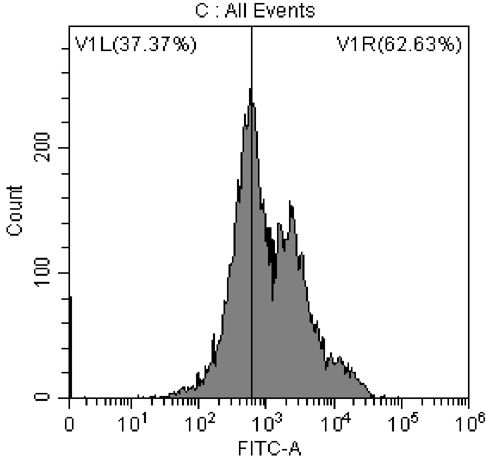

Hyp

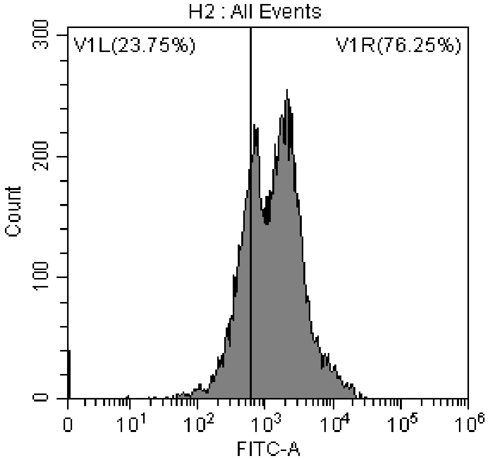

Flow Cytometry

Lysotracker (*Ex-Vivo*)

Hyp

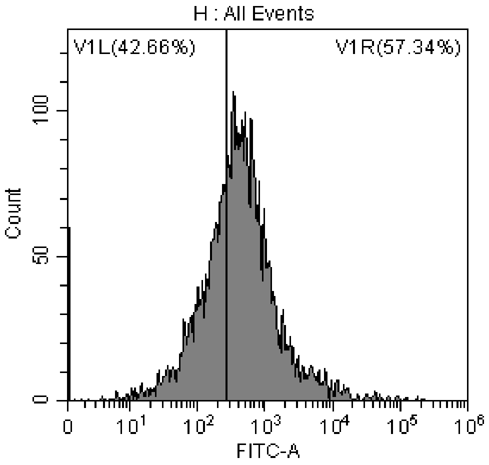

Hyp+CQ

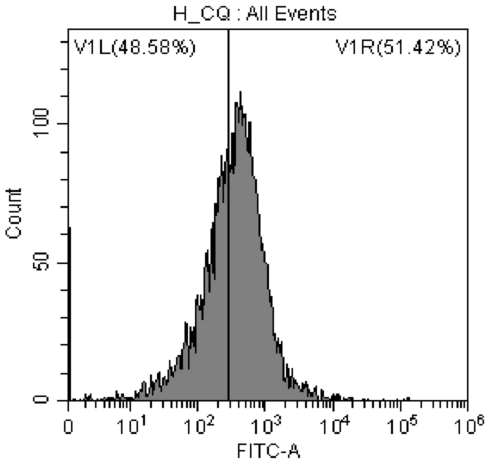

Hyp

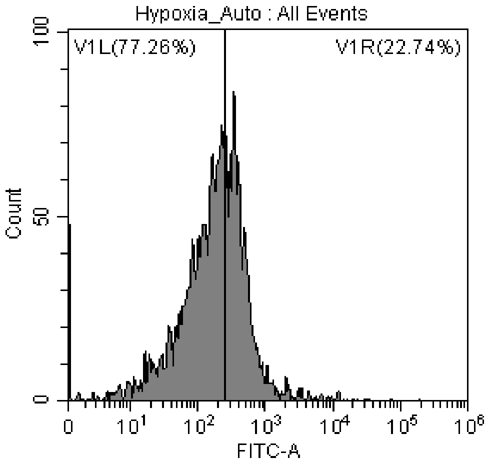

Hyp+CQ

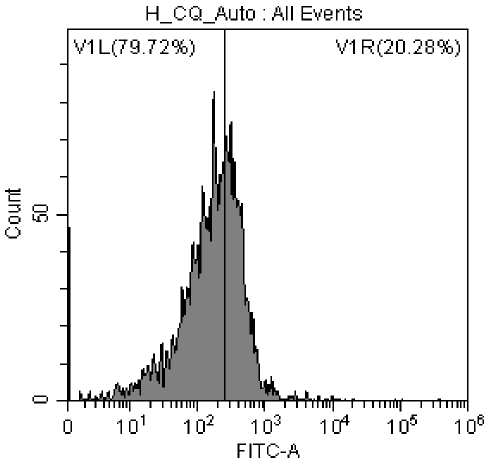

Hyp

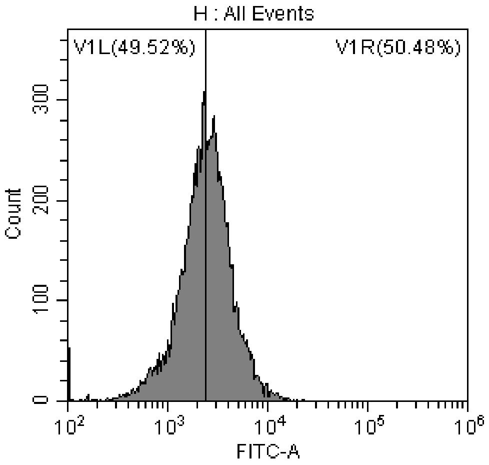

Hyp+CQ

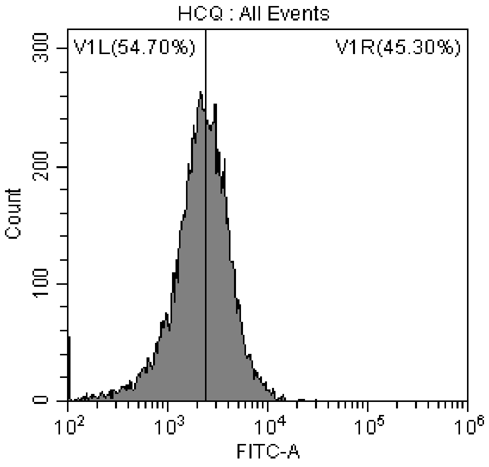

Flow Cytometry

P-Selectin (*Ex-Vivo*)

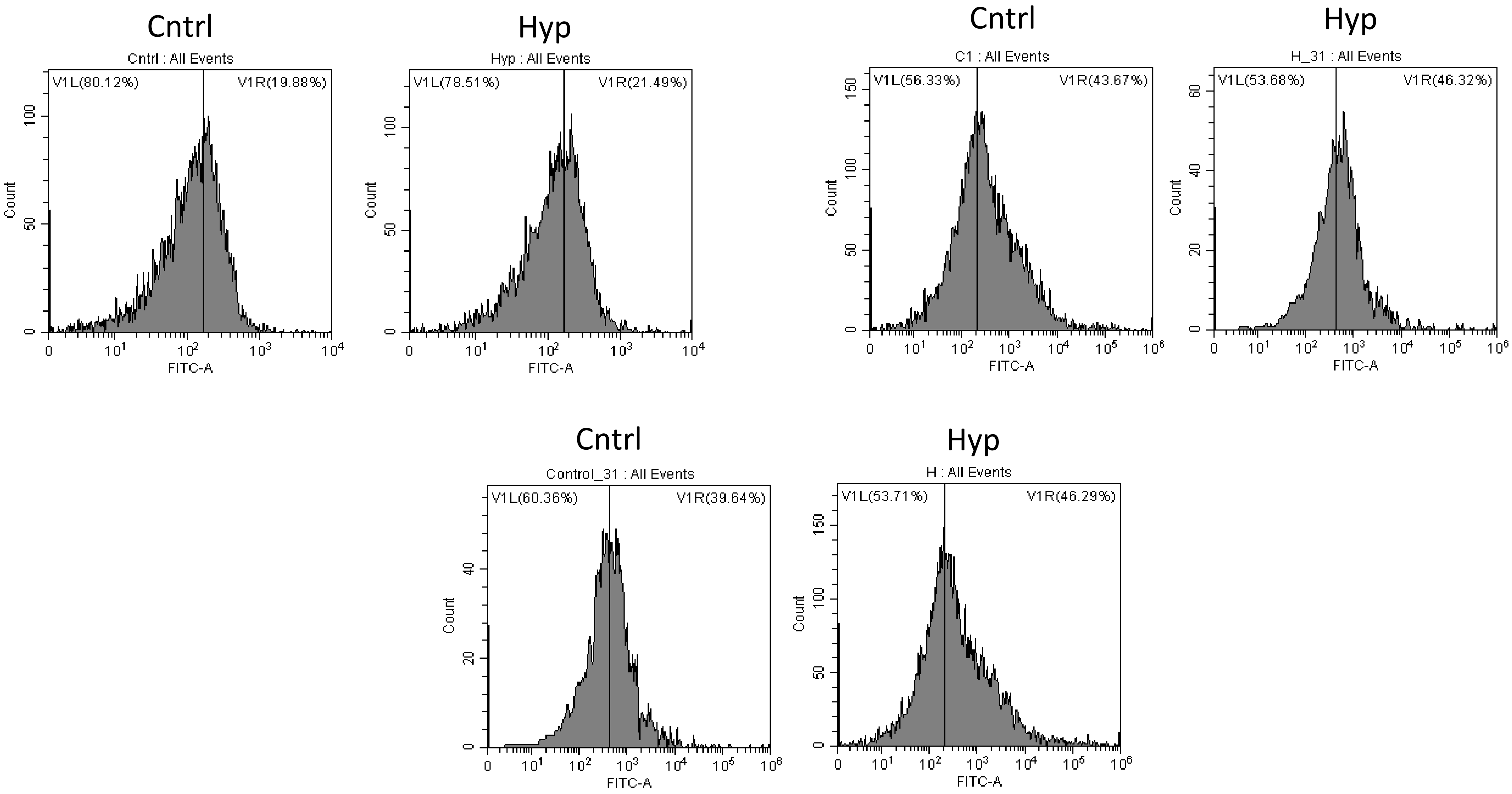

Flow Cytometry

P-Selectin (*Ex-Vivo*)

Hyp

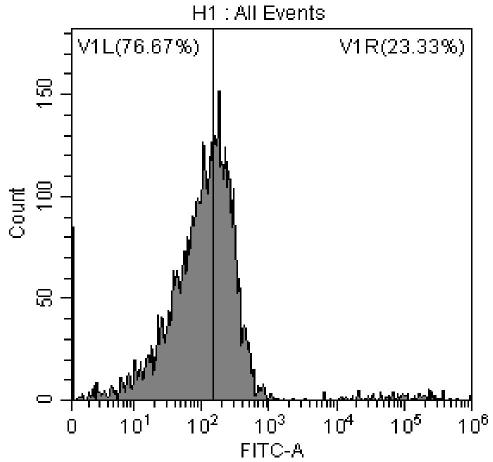

Hyp+CQ

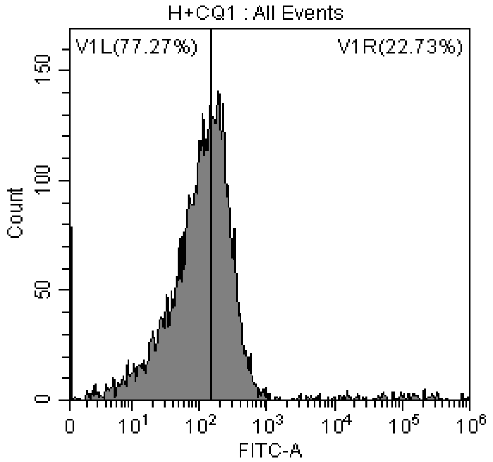

Hyp

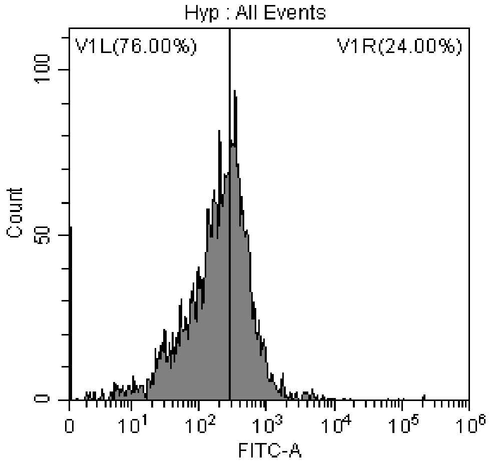

Hyp+CQ

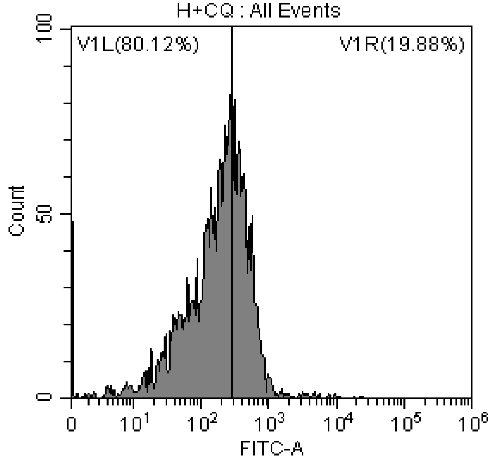

Hyp

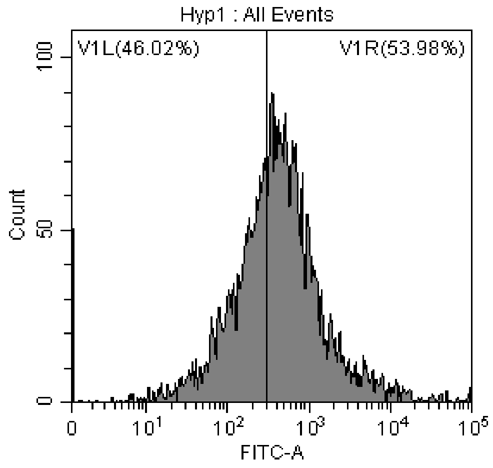

Hyp+CQ

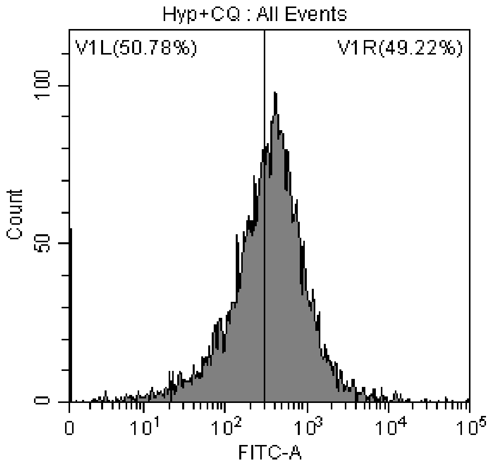

Flow Cytometry

P-Selectin (*In-Vivo*)

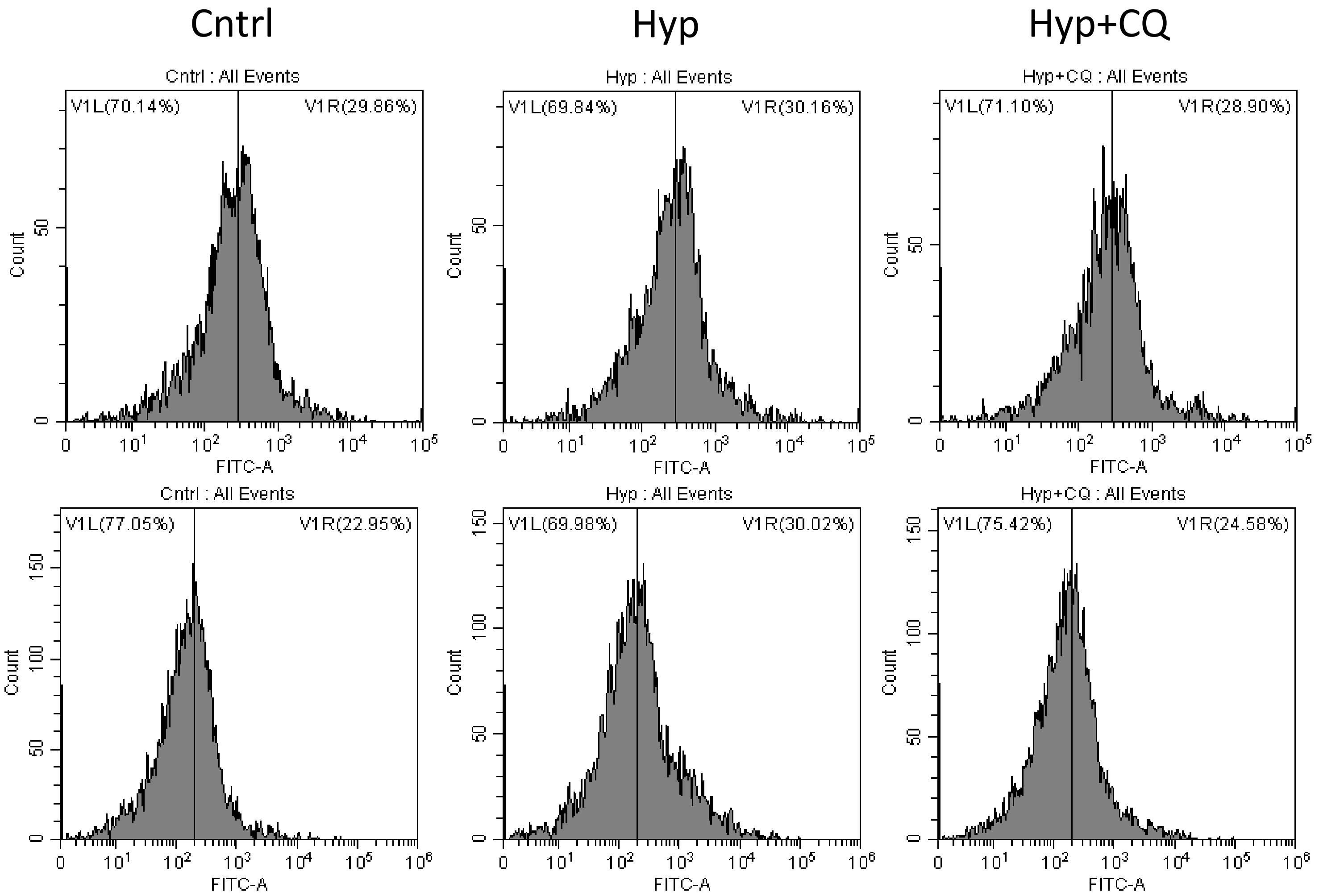

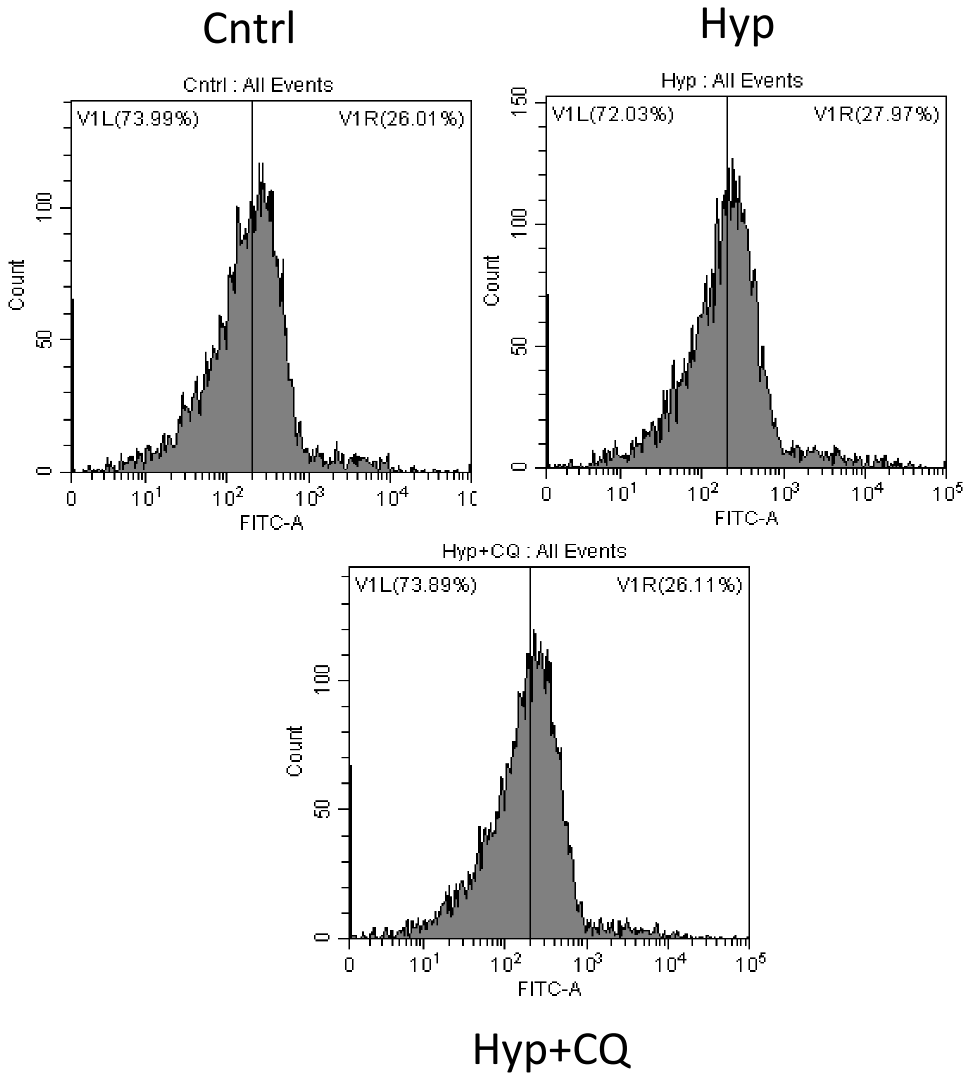

Supplement: Supplementary file 1 — Supplementary Information 1. [file 41598_2025_91181_MOESM1_ESM.pdf]
