# Supplementary material for: Inhibition of autophagy in platelets as a therapeutic strategy preventing hypoxia induced thrombosis
Source: Sci Rep. 2025 Feb 26;15:6855. doi: 10.1038/s41598-025-91181-y (PMC11865581; doi:10.1038/s41598-025-91181-y)
Supplement: Supplementary file 2 — Supplementary Information 2. [file 41598_2025_91181_MOESM2_ESM.docx]

**
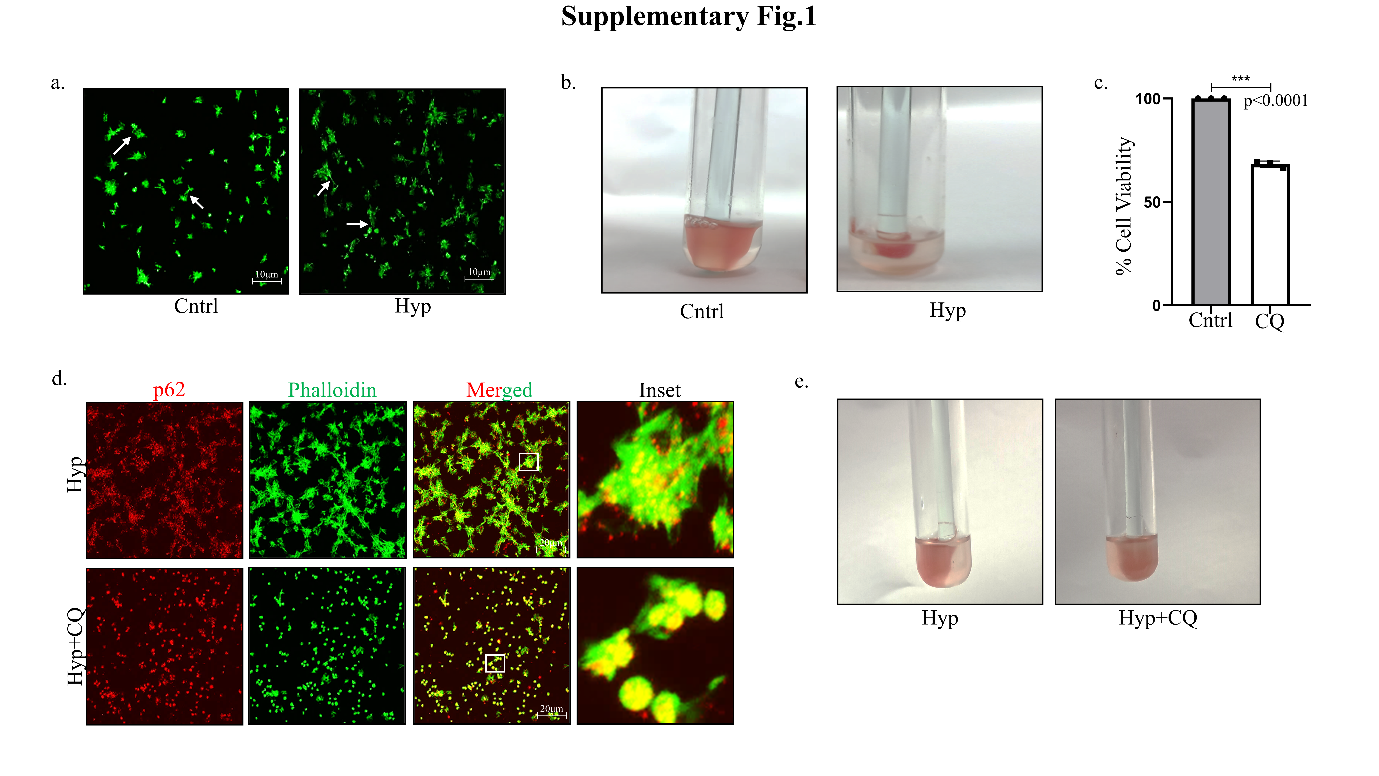
Supplementary Figures and Figure Legends**

**Supplementary figure 1:** (a) Static adhesion and platelet spreading assay showing adhesiveness of phalloidin stained platelets on collagen-coated coverslips under normoxic (Cntrl; 21% oxygen) or hypoxic (Hyp; 10% oxygen) conditions. (b) Pictorial representation of clot retraction assay under treatment conditions of 21% oxygen (Cntrl) or 10% oxygen (Hyp). (c) Chloroquine (CQ) dosing in platelets under 21% oxygen (Cntrl) condition measured by MTT assay. (d) Immunofluorescence of p62 protein (Red) in platelets treated with CQ under hypoxic condition (10% oxygen). Cells were co-stained with phalloidin (Green) (Scale bar: 20μm). **(e)** Pictorial representation of clot retraction assay performed post-CQ treatment in platelets under hypoxic condition (10% oxygen). ***p<0.001.


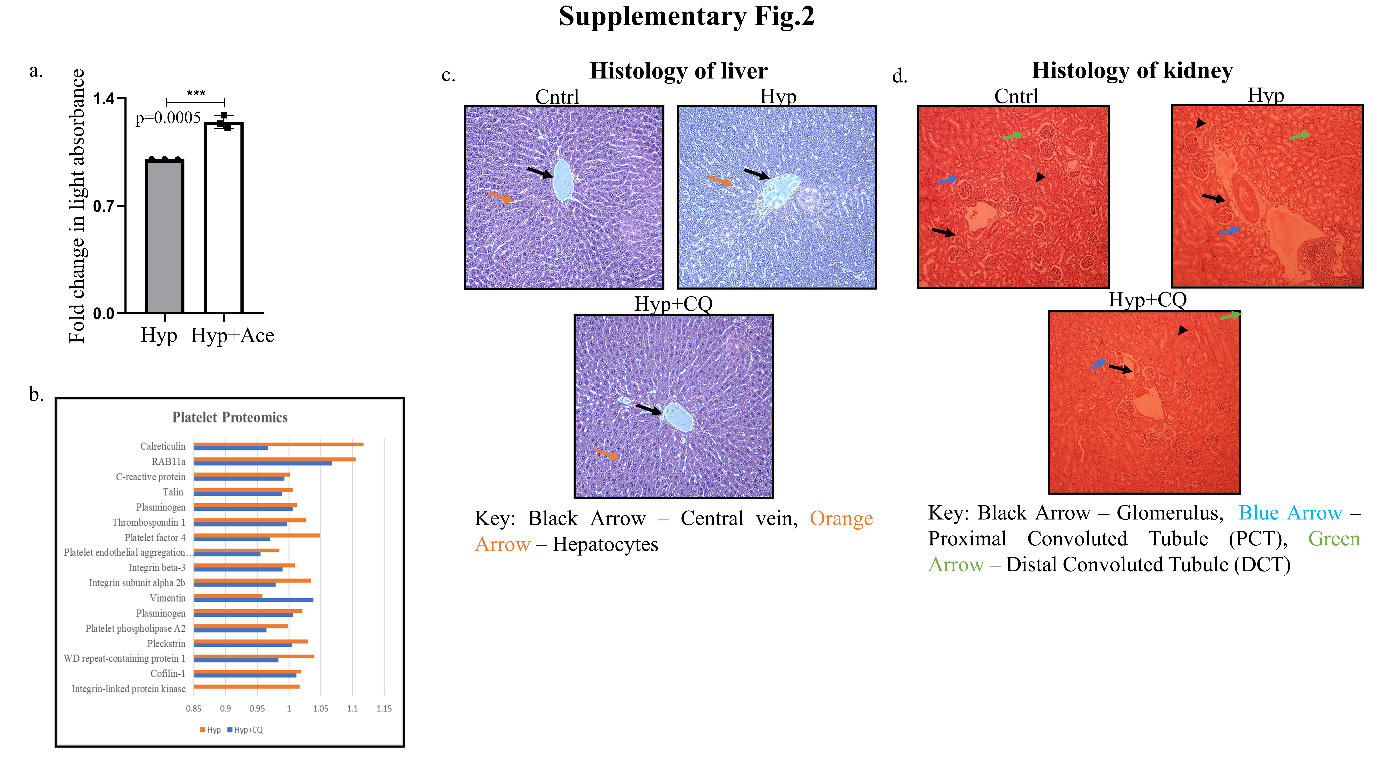


**Supplementary figure 2: (a)** Graphical representation of light absorbed by platelets post exposure to hypoxia (Hyp) with or without treatment with acetazolamide (Hyp+Ace; 100μM). **(b)** Bar graph of platelet proteomic analysis using mass spectrometry of isolated platelets obtained from animals treated with 10% oxygen (Hyp) or 10% oxygen along with CQ (Hyp+CQ) for 24hours. **(c)** Histology of liver tissue collected from animals subjected to normoxia (Cntrl, 21% oxygen) or hypoxia (Hyp, 10% oxygen) plus CQ-treated animals (Hyp+CQ). The images are labelled with black arrow for central vein and orange arrow for hepatocytes. **(d)** Histology of kidney obtained from animals post normoxia (Cntrl, 21% oxygen), hypoxia (Hyp, 10% oxygen) and CQ along with hypoxia exposure (Hyp+CQ). Images are marked with black arrow for glomerulus, blue arrow depicting proximal convoluted tubule (PCT) and green arrow showing distal convoluted tubule (DCT). ***p<0.001.
