# Supplementary material for: Inhibition of autophagy in platelets as a therapeutic strategy preventing hypoxia induced thrombosis
Source: Sci Rep. 2025 Feb 26;15:6855. doi: 10.1038/s41598-025-91181-y (PMC11865581; doi:10.1038/s41598-025-91181-y)

Supplementary Fig.3 - Original files of Fig.1

a. Cell: Platelets, Treatment: Hypoxia
Time: 30minutes Expt: Phase Contrast

Cntrl **Hyp**

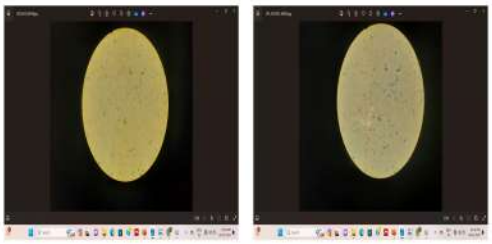

b. Cell: Platelets, Treatment: Hypoxia
Time: 30minutes Expt: LTA

Cntrl **Hyp**

	Group A	Group B
	Data Set-A	Data Set-B
1	1	0.923221
2	1	0.903882
3	1	0.934071

c. Cell: Platelets, Treatment: Hypoxia
Time: 30minutes Expt: SEM

Cntrl **Hyp**

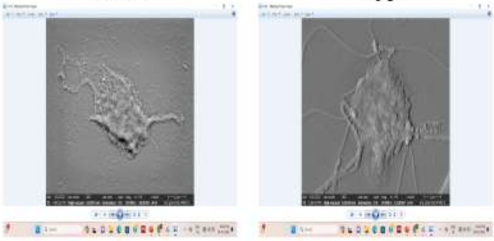

d. Cell: Platelets, Treatment: Hypoxia
Time: 30minutes Expt: Phalloidin Imaging

Cntrl **Hyp**

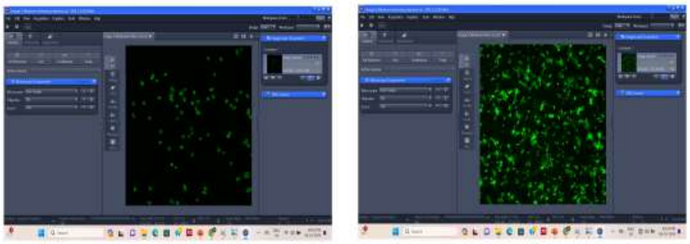

e. Cell: Platelets, Treatment: Hypoxia
Time: 30minutes Expt: P-Selectin Flow Cytometry

Link to raw file

Cntrl

Tube Name: Cntrl
Sample ID:

Population	Events	% Total	% Parent	Parent Population	Mean FITC-A
All Events	10000	100.00%	100.00%	####	31.0
P1	2127	21.27%	21.27%	All Events	47.6
Q1-UR	361	3.61%	16.97%	P1	373.3
Q1-UL	427	4.27%	20.08%	P1	232.2
Q1-LL	884	8.84%	41.56%	P1	-118.3
Q1-LR	455	4.55%	21.39%	P1	-62.0

Hyp

Tube Name: Hyp
Sample ID:

Population	Events	% Total	% Parent	Parent Population	Mean FITC-A
All Events	10000	100.00%	100.00%	####	98.9
P1	719	7.19%	7.19%	All Events	166.2
Q1-UR	178	1.78%	24.76%	P1	574.6
Q1-UL	165	1.65%	22.95%	P1	291.2
Q1-LL	247	2.47%	34.35%	P1	-95.9
Q1-LR	129	1.29%	17.94%	P1	-55.4

Supplementary Fig.4 - Original files of Fig.2

a. Cell: Platelets, Treatment: Hypoxia
Time: 30minutes Expt: Western Blotting
Lane Sequence: Cntrl Hyp Hyp+CQ CQ

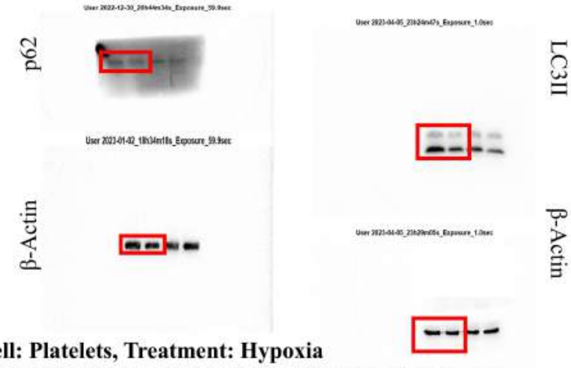

b. Cell: Platelets, Treatment: Hypoxia
Time: 30minutes Expt: IF, p62

Cntrl **Hyp**

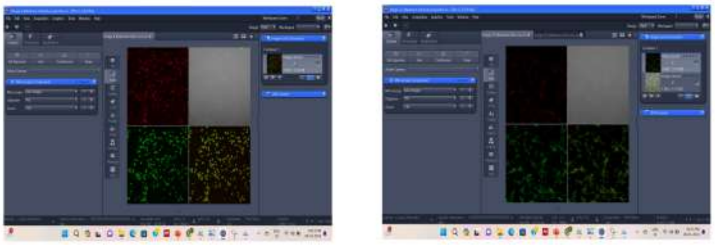

c. Cell: Platelets, Treatment: Hypoxia
Time: 30minutes Expt: Lysotracker, Flow Cytometry

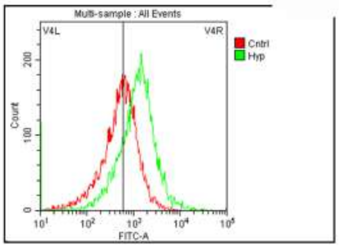

d. Cell: Platelets, Treatment: Hypoxia
Time: 30minutes Expt: IF, LAMP1

Cntrl **Hyp**

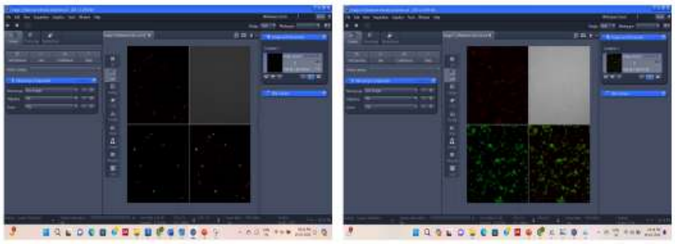

e. Cell: Platelets, Treatment: Hypoxia
Time: 30minutes Expt: Western Blotting
Lane Sequence: Cntrl Hyp Hyp+CQ CQ

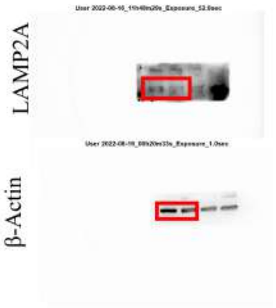

Supplementary Fig.5 - Original blots of Fig.2

a. Cell: Platelets, Treatment: Hypoxia
Time: 30minutes Expt: Western Blotting
Lane Sequence: Cntrl Hyp Hyp+CQ CQ

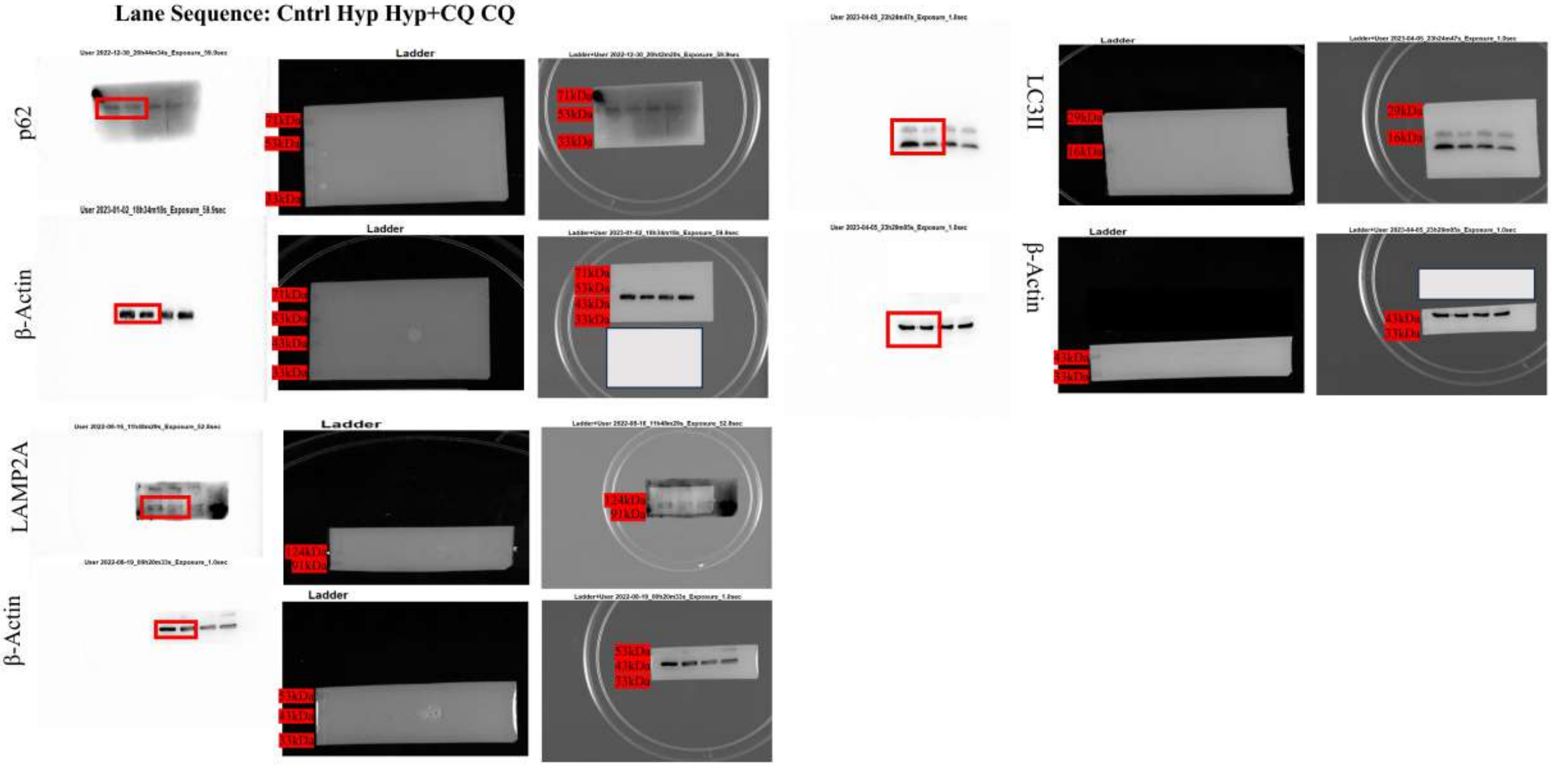

Supplementary Fig.6 - Original files of Fig.3

a. Cell: Platelets, Treatment: Hypoxia, Hypoxia+CQ
Time: 30minutes Expt: Western Blotting
Lane Sequence: Cntrl Hyp Hyp+CQ CQ

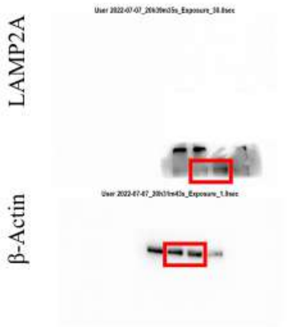

b. Cell: Platelets, Treatment: Hypoxia, Hypoxia+CQ
Time: 30minutes Expt: Western Blotting
Lane Sequence: Cntrl Hyp Hyp+CQ CQ

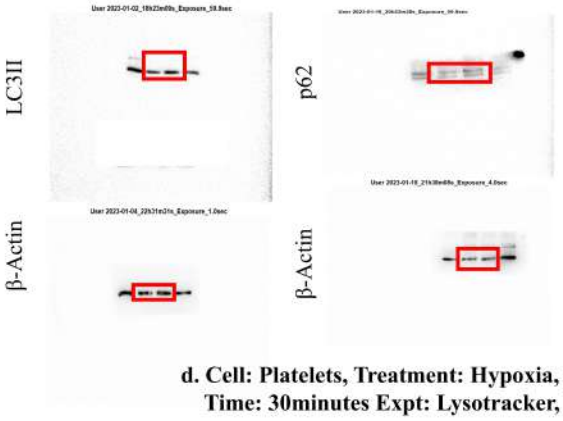

c. Cell: Platelets, Treatment: Hypoxia, Hypoxia+CQ
Time: 30minutes Expt: IF,LAMP1

Hyp

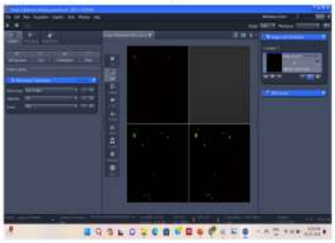

Hyp+CQ

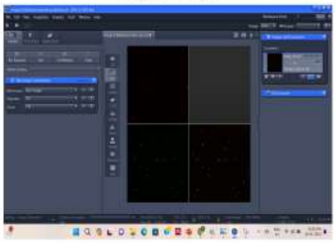

d. Cell: Platelets, Treatment: Hypoxia, Hypoxia+CQ
Time: 30minutes Expt: Lysotracker, Flow Cytometry

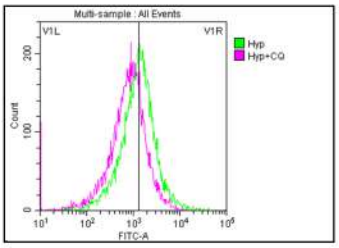

Supplementary Fig.7 - Original blots of Fig.3

a. Cell: Platelets, Treatment: Hypoxia, Hypoxia+CQ
Time: 30minutes Expt: Western Blotting
Lane Sequence: Cntrl Hyp Hyp+CQ CQ

b. Cell: Platelets, Treatment: Hypoxia, Hypoxia+CQ
Time: 30minutes Expt: Western Blotting
Lane Sequence: Cntrl Hyp Hyp+CQ CQ

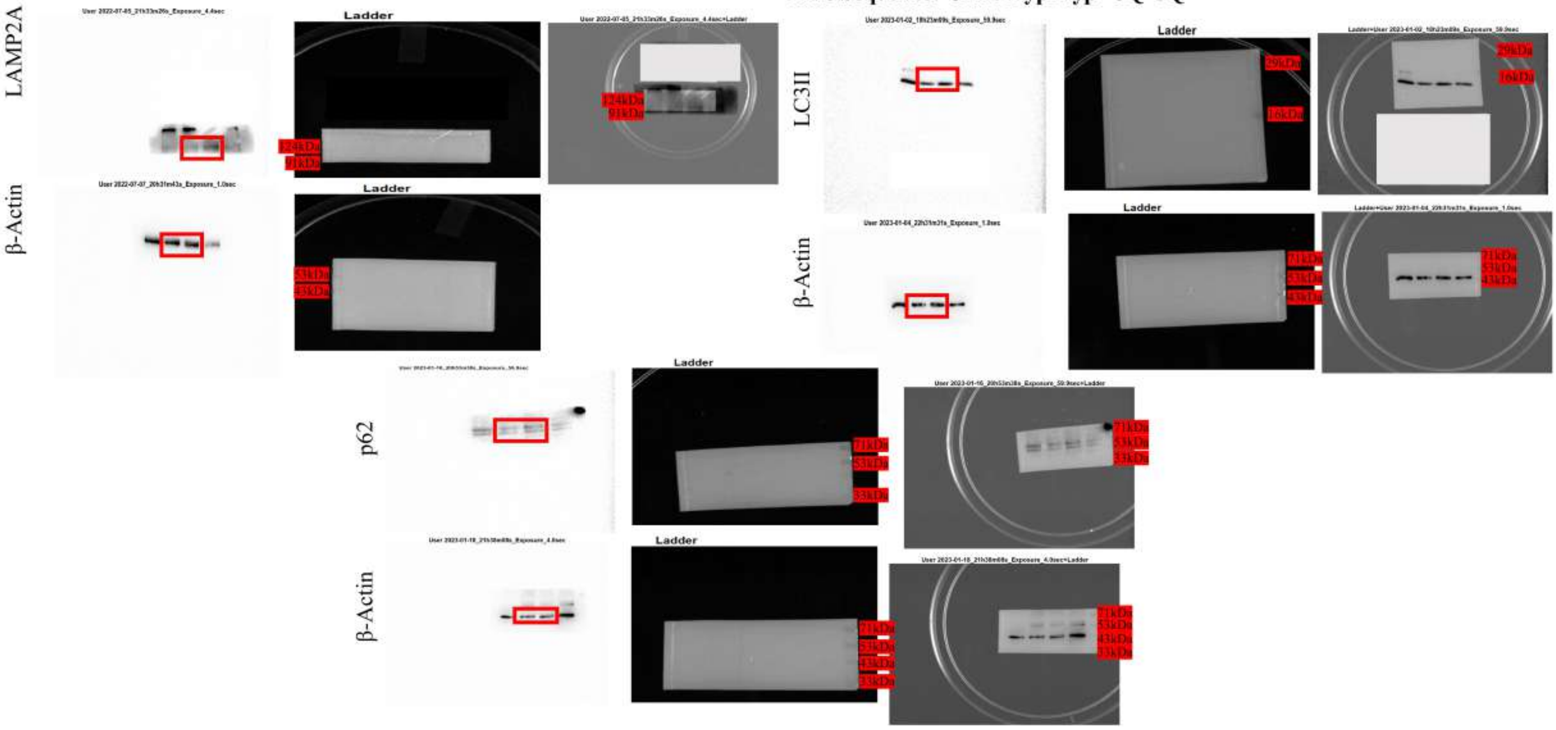

Supplementary Fig.8 - Original files of Fig.4

a. Cell: Platelets, Treatment: Hypoxia, Hypoxia+CQ
Time: 30minutes Expt: Phase Contrast

Hyp

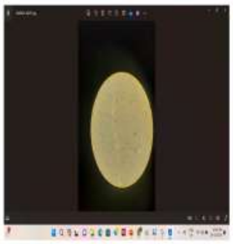

Hyp+CQ

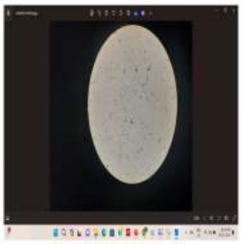

b. Cell: Platelets, Treatment: Hypoxia, Hypoxia +CQ
Time: 30minutes Expt: LTA

	Hyp	Hyp+CQ
	Group A	Group B
	Data Set-A	Data Set-B
1	1.000000	1.302658
2	1.000000	1.283976
3	1.000000	1.234375

d. Cell: Platelets, Treatment: Hypoxia, Hypoxia+CQ
Time: 30minutes Expt: SEM

Hyp

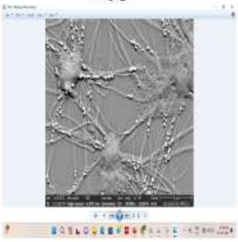

Hyp+CQ

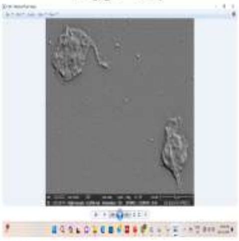

c. Cell: Platelets, Treatment: Hypoxia, Hypoxia+CQ
Time: 30minutes Expt: Phalloidin

Hyp

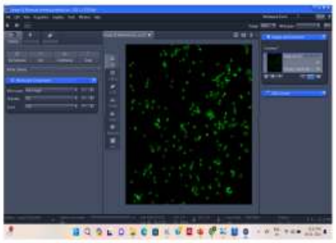

Hyp+CQ

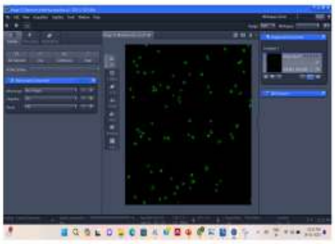

e. Cell: Platelets, Treatment: Hypoxia, Hypoxia+CQ
Time: 30minutes Expt: P-Selectin Flow Cytometry

Hyp

Tube Name: Hyp
Sample ID:

Population	Events	% Total	% Parent	Parent Population	Mean FITC-A
All Events	10000	100.00%	100.00%	####	7438.0
V1L	6176	61.76%	61.76%	All Events	-40.2
V1R	3824	38.24%	38.24%	All Events	19515.9

Hyp+CQ

Tube Name: Hyp+CQ
Sample ID:

Population	Events	% Total	% Parent	Parent Population	Mean FITC-A
All Events	10000	100.00%	100.00%	####	4427.7
V1L	6295	62.95%	62.95%	All Events	-42.6
V1R	3705	37.05%	37.05%	All Events	12023.0

Supplementary Fig.9 - Original files of Fig.5

a. Animals, Treatment: Hypoxia, Hypoxia+CQ
Time: 24 hours Expt: Tail bleeding time

	Hyp	Hyp+CQ
	Group A	Group B
	)ata Set-/	)ata Set-I
1	74	174
2	80	162
3	82	157

b. Animals, Treatment: Hypoxia, Hypoxia+CQ
Time: 24 hours Expt: Tail bleeding volume

	Hyp	Hyp+CQ
	Group A	Group B(
	)ata Set-/	)ata Set-(
1	90	180
2	100	175
3	95	170

c. Animals, Treatment: Hypoxia, Hypoxia+CQ
Time: 24 hours Expt: LTA

	Hyp	Hyp+CQ
	Group A	Group B
	Data Set-A	Data Set-B
1	1.000000	1.371128
2	0.998106	1.369318
3	0.999376	1.343945

d. Animals, Treatment: Hypoxia, Hypoxia+CQ
Time: 24 hours Expt: P-Selectin, Flow Cytometry

Hyp

Tube Name: Hyp
Sample ID:

Population	Events	% Total	% Parent	Parent Population	Mean FITC-A
All Events	5000	100.00%	100.00%	####	407.0

Hyp+CQ

Tube Name: Hyp+CQ
Sample ID:

Population	Events	% Total	% Parent	Parent Population	Mean FITC-A
All Events	5000	100.00%	100.00%	####	202.8

e. Animals , Treatment: Hypoxia, Hypoxia+CQ
Time: 24 hours Expt: Western Blotting
Lane Sequence: Cntrl Hyp Hyp+CQ CQ

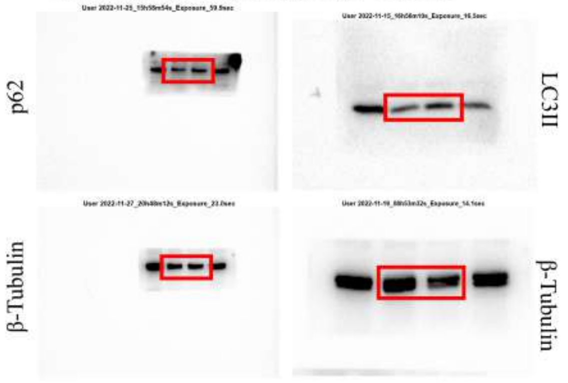

Supplementary Fig.10 - Original blots of Fig.5

e. Animals , Treatment: Hypoxia, Hypoxia+CQ
Time: 24 hours Expt: Western Blotting
Lane Sequence: Cntrl Hyp Hyp+CQ CQ

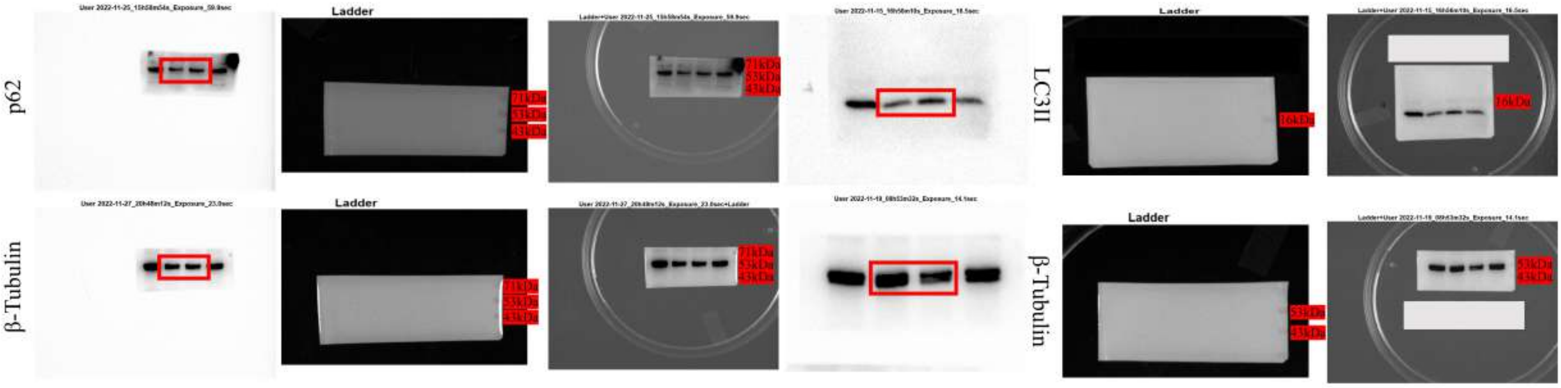

Supplementary Fig.11 - Original files of Fig.6

c. Animals, Treatment: CQ
Time: 24 hours Expt: IVC ligation, Thrombus Length

	Cntrl	CQ
	Group A	Group B
	ata Set-	ata Set-
1	1.7	1.0
2	1.5	1.2
3	1.2	0.8

d. Animals, Treatment: CQ
Time: 24 hours Expt: IVC ligation, Thrombus Weight

	Cntrl	CQ
	Group A	Group B
	Data Set-A	ata Set-
1	222.0000	94
2	232.0000	104
3	207.0000	128

e. Animals, Treatment: CQ
Time: 24 hours Expt: IVC ligation, H&E stain thrombus

Cntrl

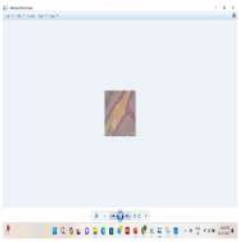

CQ

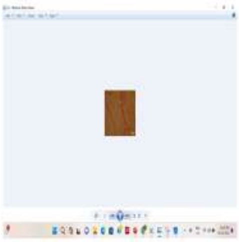

Supplementary Fig.12 – Original files of Supplementary Figure 1

a. Cells: Platelets, Treatment: Hypoxia
Time: 30minutes Expt: Static adhesion and platelet spreading assay

Cntrl **Hyp**

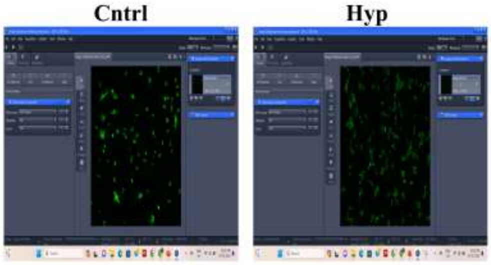

b. Cells: Platelets, Treatment: Hypoxia
Time: 30minutes Expt: Clot retraction assay

Cntrl **Hyp**

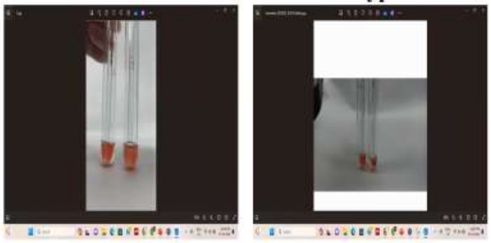

c. Cells: Platelets, Treatment: CQ
Time: 30minutes Expt: MTT Assay

	Cntrl	CQ
	Group A	Group B
	Data Set-A	Data Set-B
1	100.00000000	69.36877000
2	100.00000000	66.39478000
3	100.00000000	68.86415525

d. Cells: Platelets, Treatment: Hypoxia, Hypoxia+CQ
Time: 30minutes Expt: Immunofluorescence of p62

Hyp **Hyp+CQ**

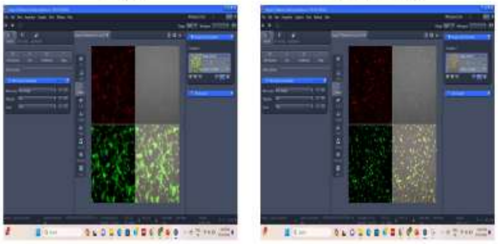

e. Cells: Platelets, Treatment: Hypoxia, Hypoxia+CQ
Time: 30minutes Expt: Clot retraction assay

Hyp **Hyp+CQ**

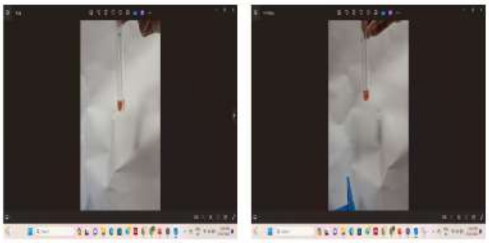

Supplementary Fig.13 - Original files of Supplementary Figure 2

a. Cell: Platelets,
Treatment:
Hypoxia, Hypoxia + Acetazolamide
Time: 30minutes
Expt: LTA

	Group A	Group B
	Data Set-A	Data Set-B
1	1.001099	1.234066
2	1.002545	1.208651
3	1.000847	1.289831
4		

c. Animals, Treatment: Normoxia (Cntrl), Hypoxia (Hyp), Hypoxia+CQ (Hyp+CQ)
Time: 24 hours Expt: Histology of Liver

Cntrl

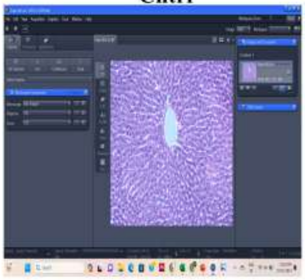

Hyp

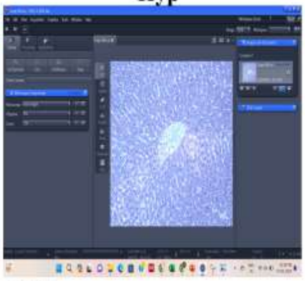

Hyp+CQ

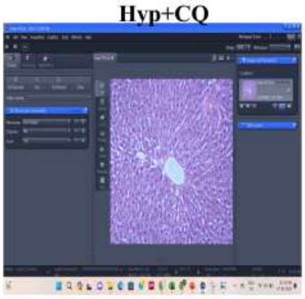

d. Animals, Treatment: Normoxia (Cntrl), Hypoxia (Hyp), Hypoxia+CQ (Hyp+CQ)
Time: 24 hours Expt: Histology of Kidney

Cntrl

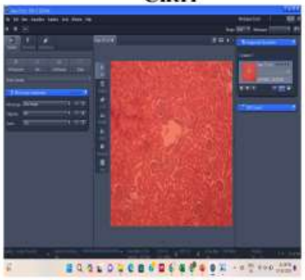

Hyp

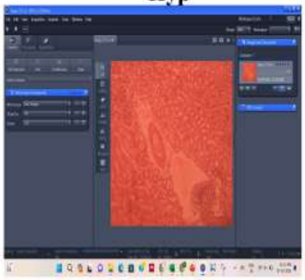

Hyp+CQ

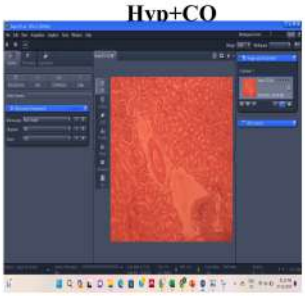

Supplement: Supplementary file 3 — Supplementary Information 3. [file 41598_2025_91181_MOESM3_ESM.pdf]
